# Loss of the *Drosophila *cell polarity regulator Scribbled promotes epithelial tissue overgrowth and cooperation with oncogenic Ras-Raf through impaired Hippo pathway signaling

**DOI:** 10.1186/1471-213X-11-57

**Published:** 2011-09-29

**Authors:** Karen Doggett, Felix A Grusche, Helena E Richardson, Anthony M Brumby

**Affiliations:** 1 Cell cycle and development laboratory. Peter MacCallum Cancer Centre, 7 St Andrews Place, East Melbourne, 3002, Victoria, Australia; 2 Colon biology laboratory. Ludwig Institute for Cancer Research, Royal Parade, Parkville, 3052, Victoria, Australia; 3 Anatomy and Cell Biology Department, University of Melbourne, Melbourne, 3010, Victoria, Australia; 4 Cell growth and proliferation laboratory. Peter MacCallum Cancer Centre, 7 St Andrews Place, East Melbourne, 3002, Victoria, Australia; 5 Biochemistry and Molecular Biology Department, University of Melbourne, Melbourne, 3010, Victoria, Australia

## Abstract

**Background:**

Epithelial neoplasias are associated with alterations in cell polarity and excessive cell proliferation, yet how these neoplastic properties are related to one another is still poorly understood. The study of *Drosophila *genes that function as neoplastic tumor suppressors by regulating both of these properties has significant potential to clarify this relationship.

**Results:**

Here we show in *Drosophila *that loss of Scribbled (Scrib), a cell polarity regulator and neoplastic tumor suppressor, results in impaired Hippo pathway signaling in the epithelial tissues of both the eye and wing imaginal disc. *scrib *mutant tissue overgrowth, but not the loss of cell polarity, is dependent upon defective Hippo signaling and can be rescued by knockdown of either the TEAD/TEF family transcription factor Scalloped or the transcriptional coactivator Yorkie in the eye disc, or reducing levels of Yorkie in the wing disc. Furthermore, loss of Scrib sensitizes tissue to transformation by oncogenic Ras-Raf signaling, and Yorkie-Scalloped activity is required to promote this cooperative tumor overgrowth. The inhibition of Hippo signaling in *scrib *mutant eye disc clones is not dependent upon JNK activity, but can be significantly rescued by reducing aPKC kinase activity, and ectopic aPKC activity is sufficient to impair Hippo signaling in the eye disc, even when JNK signaling is blocked. In contrast, *warts *mutant overgrowth does not require aPKC activity. Moreover, reducing endogenous levels of aPKC or increasing Scrib or Lethal giant larvae levels does not promote increased Hippo signaling, suggesting that aPKC activity is not normally rate limiting for Hippo pathway activity. Epistasis experiments suggest that Hippo pathway inhibition in *scrib *mutants occurs, at least in part, downstream or in parallel to both the Expanded and Fat arms of Hippo pathway regulation.

**Conclusions:**

Loss of Scrib promotes Yorkie/Scalloped-dependent epithelial tissue overgrowth, and this is also important for driving cooperative tumor overgrowth with oncogenic Ras-Raf signaling. Whether this is also the case in human cancers now warrants investigation since the cell polarity function of Scrib and its capacity to restrain oncogene-mediated transformation, as well as the tissue growth control function of the Hippo pathway, are conserved in mammals.

## Background

*Drosophila *has long been recognized as an important model organism for elucidating oncogenic and tumor suppressor pathways [reviewed in [1]]. Traditionally two distinct classes of tumor suppressor mutants have been described, the loss of which cause either hyperplastic or neoplastic overgrowth [reviewed in [2]]. Hyperplastic overgrowth is characterized by excessive cell proliferation that is eventually restrained by terminal differentiation, while neoplastic overgrowth exhibits impaired differentiation, defects in cell polarity and the propensity to invade and metastasize.

Over recent years, a large number of hyperplastic tumor suppressor mutants have been united into a single pathway, the Hippo pathway [reviewed in [3]]. Core components of the Hippo pathway include the serine-threonine kinases Hippo (Hpo) and Warts (Wts), and their adaptor proteins, Salvador (Sav) and Mob-As-Tumor-Suppressor (Mats). Hpo phosphorylates and activates Wts, and Wts phosphorylates and thereby inactivates the transcription co-factor Yorkie (Yki). Loss of Hippo pathway components leads to reduced phosphorylation of Yki and its translocation to the nucleus where it binds to its DNA binding partner, Scalloped (Sd), and promotes expression of proteins involved in cell proliferation (Cyclin E; CycE), cell growth (Myc) and cell survival (*Drosophila *Inhibitor of Apoptosis 1; DIAP1) [4-11]. It is the dual role of the Hippo pathway in regulating both cell proliferation and survival functions that makes its loss such a potent driver of tissue overgrowth. The pathway is regulated through input from upstream components including Merlin and Expanded (Ex), and the transmembrane proteins Fat (Ft) and Dachsous (Ds) [12-19]. It is proposed that the primary function of the Hippo pathway is to incorporate positional cues within an epithelial field to dictate the ultimate size of organ development [20]. The pathway is highly conserved and also functions to restrain organ size in mammals. Furthermore, increasing evidence links Hippo pathway deregulation to tumorigenesis [reviewed in [21]].

In contrast to the hyperplastic Hippo pathway mutants, mutants that result in neoplastic overgrowth are characterized by alterations in cell polarity and a failure to terminally differentiate. Neoplastic mutants include the junctional scaffolding genes that regulate cell polarity, *scrib, discs large *(*dlg*) and *lethal giant larvae *(*lgl*), as well as mutants within the endocytic pathway including *avalanche *(*avl*), *tumor suppressor protein 101 *(*TSG101*) and *Rab5 *[reviewed in [22]]. The loss of apico-basal cell polarity and overgrowth phenotypes of a number of these mutants, including *scrib, lgl, avl *and *TSG101 *are dependent upon atypical protein kinase C (aPKC) activity, since mutant phenotypes can be rescued by reducing atypical protein kinase C function [23-25]. Direct and mutual antagonism between the junctional tumor suppressors and aPKC has been demonstrated by the ability of aPKC to associate with and phosphorylate Lgl, thereby releasing Lgl from the cell cortex and thus potentially inhibiting Lgl function [26], and the ability of Lgl to inhibit aPKC-dependent phosphorylation of other key targets [27]. Like the Hippo pathway, mammalian homologues of the *Drosophila *neoplastic tumor suppressors, as well as aPKC, are increasingly implicated as important players in human cancers [reviewed in [28]].

It is now becoming apparent that these two formerly separate classes of hyperplastic and neoplastic tumor regulators are interconnected. Indeed, *wts *mutants were originally identified based upon mutant cell morphology [29], and this is now known to be a phenotype associated with other Hippo pathway mutants and due to Yki-dependent upregulation of the apical cell polarity determinant Crumbs (Crb) and apical hypertrophy [30,31]. Crb itself acts to regulate Hippo signaling by binding to Ex [32], and either excessive Crb activity or loss of Crb results in deregulation of Ex and an impairment to Hippo signaling resulting in tissue overgrowth [32-35]. The neoplastic tumor suppressors *scrib, dlg *and *lgl *also interact with the Hippo pathway. *dlg, lgl *or *scrib *mutant follicle cells surrounding the female ovary have elevated levels of Yki targets Cyclin E and DIAP1, and exhibit strong genetic interactions with *wts *[36]. Furthermore, loss of *lgl *has been shown to impair Hippo signaling in the eye disc in an aPKC signaling-dependent manner [35]. Indeed in the wing disc both the loss of *lgl *or ectopic aPKC activity promotes Yki activity through an upregulation of Jun N-terminal kinase (JNK) signaling [37]. Whether *scrib *regulates the Hippo pathway in the eye or wing disc is not yet clear. In contrast to *lgl*, reduced levels of Scrib in the eye disc to levels where apico-basal cell polarity is only mildly affected does not impair Hippo signaling [35], although prior studies with null alleles have demonstrated both aPKC-dependent proliferation as well as ectopic JNK signaling in *scrib *mutant eye disc clones [38]. Furthermore, whilst homozygous *scrib *mutant wing disc overgrowth is reduced in response to limiting Yki levels [35], it has not been determined if Hippo signaling is impaired in this tissue and whether the genetic interaction with *yki *reflects a general sensitivity of *scrib *mutant tissue to limiting levels of survival/proliferation functions. Clarifying the relationship between *scrib *and the Hippo pathway is therefore required.

In this study, we show that loss of *scrib* promotes eye and wing disc epithelial tissue overgrowth, as well as cooperative neoplastic overgrowth with oncogenic Ras-Raf signaling, through impaired Hippo pathway signaling. Significantly, despite JNK signaling being activated in the absence of *scrib*, the Hippo pathway remains impaired even when JNK is blocked. In contrast, Hippo pathway deregulation in *scrib *mutants is dependent upon aPKC signaling, and ectopic activation of aPKC is sufficient to downregulate the Hippo pathway independent of JNK signaling. As both the Scribble cell polarity module and the Hippo pathway are conserved in mammals, and loss of apico-basal cell polarity is a hallmark of mammalian epithelial neoplasias, it is likely that our results have significant implications for human tumorigenesis.

## Results

### *scrib *mutant eye disc cells exhibit impaired Hippo pathway signaling, and this does not require JNK signaling

We have previously reported that *scrib *mutant clones of tissue in the eye disc exhibit ectopic CycE expression and excessive cell proliferation, although this is restrained through JNK-dependent apoptosis [39]. If JNK signaling is blocked in *scrib *mutant clones by expressing a dominant negative version of *Drosophila *JNK, *bsk *(*bsk^DN^*), apoptosis is prevented and mutant cells are observed to ectopically proliferate posterior to the morphogenetic furrow (MF) resulting in clonal overgrowth [38]. The ectopic cell proliferation in *scrib *mutant clones expressing *bsk^DN ^*is similar to mutants in the Hippo pathway, and therefore to determine if the Hippo pathway is impaired by the loss of *scrib*, we examined known targets of Hippo-mediated repression in *scrib *mutant eye disc clones. Targets examined included protein levels of DIAP1 [40], and expression of the enhancer traps for *four-jointed *(*fj-lacZ*) and *ex *(*ex-lacZ*), both of which are known to function as readouts of impaired Hippo signaling [17,41].

**Figure 1:**
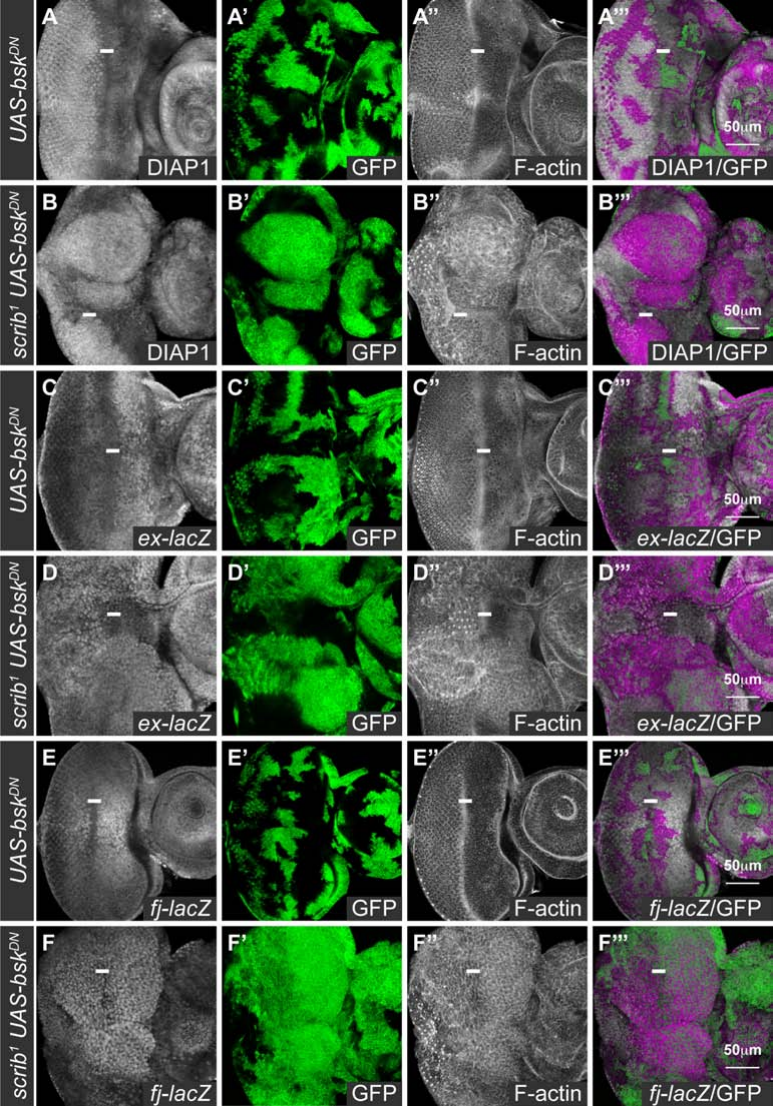
***scrib *mutant cells expressing *bsk^DN ^*in the eye disc have impaired Hippo pathway signaling**. Confocal sections through 3^rd ^instar larval eye/antennal discs (A-F), posterior to the left in this and all subsequent figures. *bsk^DN^*-expressing clones (A, C, E), and *scrib^1 ^*clones expressing *bsk^DN ^*(B, D, F) were generated with *ey-FLP *and are positively marked by GFP expression (green, or magenta in the merges). Grayscale is DIAP1 (A-B), β-GAL (C-F), and F-actin to show tissue morphology (A-F). A white bar indicates the location of the morphogenetic furrow (MF) where cells are G1-phase arrested. (A, B) DIAP1 is expressed throughout the larval eye/antennal disc, and levels of the protein increase posterior to the MF in the differentiating portion of the eye disc. *bsk^DN^*-expressing clones exhibit normal levels of DIAP1 (A), however, levels of DIAP1 are increased in *scrib^1 ^*clones expressing *bsk^DN ^*(B). (C-F) *ex-lacZ *is expressed throughout the eye/antennal disc and is higher anterior to the MF in the eye disc; and *fj-lacZ *is expressed in a gradient in the eye disc with highest levels in the anterior centre, decreasing in levels along the dorsal, ventral and posterior axes. The expression of *bsk^DN ^*does not alter *ex-lacZ *(C) or *fj-lacZ *(E) expression, however, *scrib^1 ^*clones expressing *bsk^DN ^*show elevated expression levels of *ex-lacZ *(D; note particularly clones of tissue posterior to the MF) and *fj-lacZ *(F).

The expression of Hippo pathway reporters in *scrib *mutant eye disc clones were variable, possibly due to the activation of JNK-dependent cell death pathways. However DIAP1 levels were noticeably upregulated in some mutant clones, and both the *ex-lacZ *and *fj-lacZ *reporters were also consistently upregulated in the absence of *scrib*, indicating that the Hippo pathway was being impaired (see additional file 1). As we had previously shown that *scrib *mutant cells continue to ectopically proliferate in the eye disc even when JNK signaling is blocked [38], it seemed likely that this impairment to Hippo signaling would not depend upon JNK activation. However, a previous report has highlighted the role that JNK signaling can play in promoting Hippo pathway impairment [37], and therefore we also examined the expression of the reporters in *scrib *mutant clones in which JNK signaling was blocked by the expression of *bsk^DN^*. Significantly, whilst the expression of *bsk^DN ^*alone in eye disc clones did not noticeably affect levels of DIAP1, *ex-lacZ *or *fj-lacZ *(Figure 1A, C, E), *scrib *mutant clones expressing *bsk^DN ^*had strikingly increased levels of DIAP1, as well as *ex-lacZ *and *fj-lacZ *expression (Figure 1B, D, F). Thus, Hippo pathway signaling was perturbed in both *scrib *mutant clones and *scrib *mutant clones expressing *bsk^DN^*.

The *bsk^DN ^*transgene is highly effective at blocking JNK signaling since it completely abrogates both the ectopic expression of the JNK pathway reporter, *msn-lacZ*, and the JNK pathway target, Paxillin, in *scrib *mutant clones [38]. However, to confirm that JNK signaling was not required for Hippo pathway impairment, we also knocked down *bsk *expression in *scrib *mutant cells using a *bsk^RNAi ^*transgene. Like the expression of *bsk^DN ^*in *scrib *mutant clones, this resulted in pupal lethality and in the formation of larger clones of *scrib *mutant tissue in the eye disc, suggesting that cells were no longer dying. Significantly, examination of BrdU incorporation indicated that mutant cells ectopically proliferated posterior to the MF, and also exhibited ectopic *fj-lacZ *expression (see additional file 2). Thus, whilst we do not rule out JNK-dependent effects upon the Hippo pathway in *scrib *mutants, our data indicates that the Hippo pathway is impaired in *scrib *mutant eye disc clones even when JNK signaling is blocked.

### Ectopic cell proliferation, but not the loss of apico-basal cell polarity in *scrib *mutant cells posterior to the MF, is Yki and Sd-dependent

Hippo pathway mutant defects are Yki and Sd-dependent. Loss of Yki phosphorylation results in nuclear translocation and, through association with the DNA binding protein Sd, transcriptional activation of targets including DIAP1 and CycE. Removing Yki function can rescue Hippo pathway mutant overgrowth, however, Yki is also required for normal cell proliferation in the eye disc [7]. In contrast, Sd is largely dispensable for normal eye disc growth and proliferation and specifically mediates Hippo pathway mutant overgrowth [4,5]. Therefore, to determine if the ectopic cell proliferation and altered cell morphology of *scrib *mutant cells were due to loss of Hippo pathway signaling we utilized RNAi-mediated knockdown of *sd *function in *scrib *mutant eye disc clones to look for rescue of the mutant phenotype.

**Figure 2:**
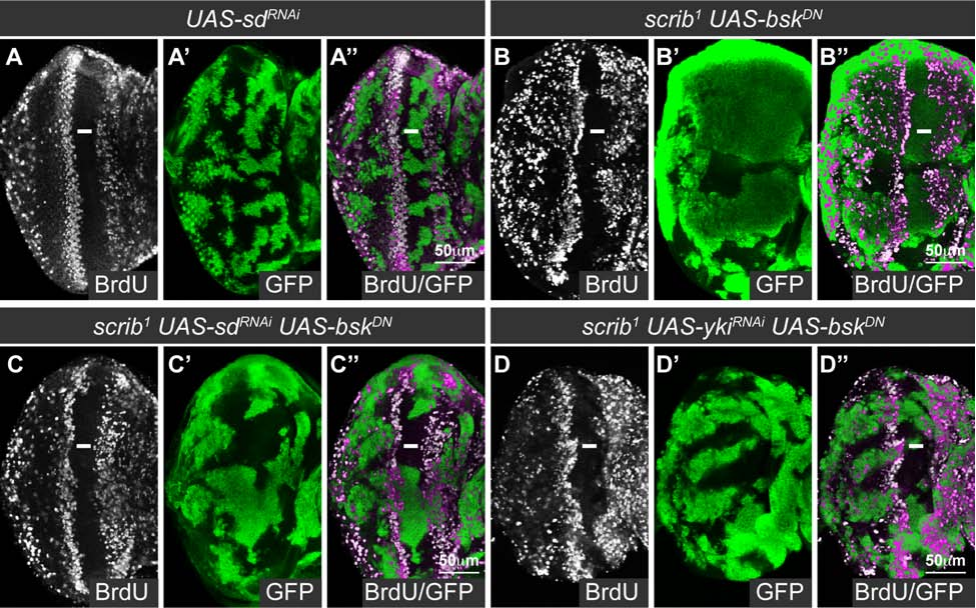
**Sd and Yki are required for the ectopic cell proliferation in *scrib *mutant eye disc clones**. Mutant eye/antennal disc clones were generated with *ey-FLP *and are positively marked by GFP expression (green, or magenta in the merges). Grayscale is BrdU. A white bar indicates the location of the MF. (A-D) RNAi-mediated knockdown of *sd *in clones does not alter the normal pattern of BrdU incorporation in the eye/antennal disc (A), whilst *scrib^1 ^*clones expressing *bsk^DN ^*are large and ectopically proliferate posterior to the MF (B). Knockdown of *sd *in *scrib^1 ^*clones expressing *bsk^DN ^*reduces the ectopic cell proliferation in the mutant clones posterior to the MF (C), and a similar rescue is shown by knockdown of *yki *(D).

Expression of *sd^RNAi ^*in otherwise wild type clones of tissue produced no discernible effect on cell viability or proliferation in larval discs (Figure 2A) and in adult eyes (data not shown). However, consistent with previous reports, when *sd^RNAi ^*was expressed in *wts^X1 ^*mutant clones it completely abrogated the hyperplasia of the mutant tissue thus confirming the efficacy of *sd *knockdown (see additional file 3). To determine if *sd^RNAi ^*could also rescue the ectopic cell proliferation in *scrib *mutant eye disc clones, we coexpressed *sd^RNAi ^*with *bsk^DN ^*in *scrib *mutant eye disc clones. Examination of BrdU incorporation showed that although *scrib *mutant clones expressing *bsk^DN ^*exhibited ectopic BrdU incorporation posterior to the MF (Figure 2B), the coexpression of *sd^RNAi ^*in the mutant tissue significantly reduced proliferation in the mutant tissue posterior to the MF (Figure 2C). Reducing *sd *function also efficiently abrogated the ectopic *fj-lacZ *expression in *scrib *mutant eye disc clones, thereby confirming that the rescue to proliferation correlated with a normalization in Hippo pathway reporter expression (see additional file 4). Furthermore, knockdown of *yki *with RNAi in *scrib *mutant clones expressing *bsk^DN ^*also significantly reduced the ectopic BrdU incorporation in *scrib *mutant cells posterior to the MF (Figure 2D), thus demonstrating that both Sd and Yki activity were required to drive ectopic cell proliferation.

**Figure 3:**
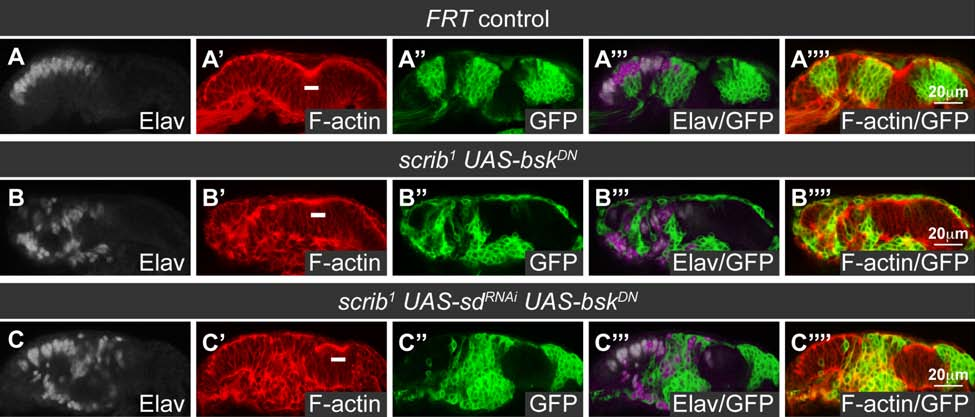
**Sd is not required for the cell morphology defects in *scrib *mutant clones**. Cross sections of larval eye discs. Mutant clones (A-C) were generated with *ey-FLP *and are positively marked by GFP expression (green, or magenta and yellow in the merges). Grayscale is Elav and red is F-actin. A white bar indicates the location of the MF. (A-C) Control eye disc clones, generated using a wild type chromosome with a *Flippase recognition target *(*FRT*), show the columnar epithelial structure of the eye disc, with the apically localized nuclei of the developing photoreceptor cells marked by Elav (A). In contrast, *scrib^1 ^*clones expressing *bsk^DN ^*show a disorganized epithelial structure resulting in Elav positive photoreceptor cell nuclei being mislocalized basally within the epithelium (B). Knockdown of *sd *in *scrib^1 ^*clones expressing *bsk^DN ^*does not rescue the mutant cell morphology defects (C).

To determine if the rescue in cell proliferation was accompanied by a restoration in mutant cell morphology, we also examined the eye disc epithelium in cross section with F-actin staining. Normally the Elav-expressing photoreceptor cells are apically localized within the pseudo-stratified columnar epithelium (Figure 3A), however, in *scrib *mutant clones, cells are often extruded basally and Elav-positive nuclei are aberrantly localized basally within the epithelium [38]. In *scrib *mutants (data not shown), or in *scrib *mutants protected from cell death by the expression of *bsk^DN^*, knockdown of *sd *function with *sd^RNAi ^*failed to restore normal cell morphology to the mutant tissue (Figure 3B, C). Furthermore, this did not reflect a Sd-independent activity of impaired Hippo signaling since halving the gene dosage of *yki*, the direct target of Hippo-mediated repression, or knockdown of *yki *with RNAi, also failed to restore normal cell morphology to the mutant tissue (data not shown). Thus, although *scrib *mutant cells ectopically proliferate due to downregulation of Hippo signaling, impaired Hippo signaling is not necessary for mediating the apico-basal cell polarity defects in *scrib *mutant tissue.

### *scrib *mutant wing disc tissue overgrows through impaired Hippo signaling

**Figure 4:**
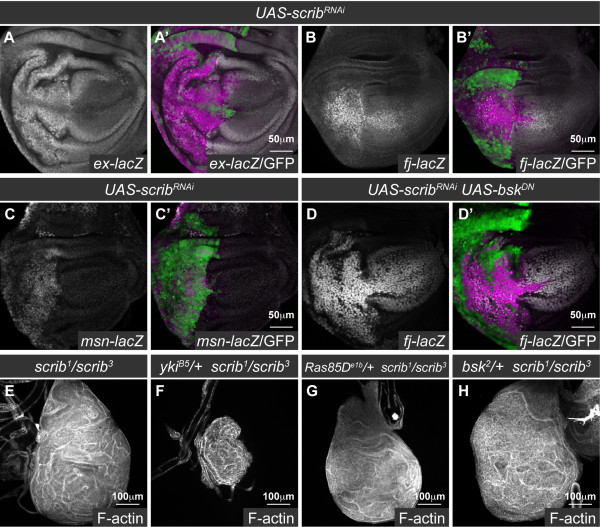
***scrib *mutant wing disc tissue overgrows through impaired Hippo signaling**. Confocal sections through 3^rd ^instar larval wing discs (A-H), posterior to the left. *en-GAL4 *driven expression of transgenes in the posterior half of the discs (A-D) is marked by GFP expression (green, or magenta in the merges). Grayscale is β-GAL (A-D) or F-actin (E-H). (A-D) *en-GAL4 *driven expression of *scrib^RNAi ^*in the posterior half of the wing disc increases *ex-lacZ *(A), *fj-lacZ *(B) and *msn-lacZ *(C) expression. Coexpression of *bsk^DN ^*with *scrib^RNAi ^*does not rescue the increased expression of *fj-lacZ *(D). (E-H) *scrib^1^/scrib^3 ^*transheterozygous mutant wing discs over grow and become increasingly disorganized (E). Halving the gene dosage of *yki *dramatically reduces the size of homozygous *scrib *mutant wing discs (F). In contrast, neither halving the gene dosage of *Ras85D *(G) or *bsk *(H) reduces homozygous *scrib *mutant wing disc overgrowth.

To determine if loss of *scrib *could also impair Hippo signaling in other epithelial imaginal tissues, we examined the larval wing disc. Using *en-GAL4 *to direct RNAi-mediated knockdown of *scrib *in the posterior half of the wing disc, we observed a decrease in Scrib protein levels and alterations in cell morphology (see additional file 5). Significantly, this was accompanied by an increase in the expression of *ex-lacZ *throughout the posterior half of the wing disc and *fj-lacZ *in the wing pouch region (Figure 4A, B), thus confirming that loss of *scrib *impairs Hippo signaling in the wing disc.

Loss of *lgl* in the wing disc also impairs Hippo signaling, and this has been shown to depend upon JNK signaling [37]. Indeed, as in the eye disc [38,39], loss of *scrib *in the wing disc similarly led to the ectopic expression of the JNK pathway reporter *msn-lacZ *(Figure 4C). However, surprisingly, blocking JNK with the expression of *bsk^DN ^*failed to normalize *fj-lacZ *expression (Figure 4D), indicating that loss of *scrib *in the wing disc also results in an impairment of Hippo pathway signaling that cannot be rescued by inhibiting JNK.

Having established that Hippo signaling was perturbed by *scrib *knockdown in the wing disc, we next wished to confirm that this was important for driving *scrib *mutant tissue overgrowth. Trans-heterozygous *scrib^1 ^*over *scrib^3 ^*larvae fail to pupate and form giant overgrown larvae, and whilst the eye discs do not noticeably overgrow and are reduced in size presumably due to increased cell death, the wing discs over-proliferate and become highly folded and irregular in appearance (Figure 4E). To determine if wing disc overgrowth in *scrib *mutants was dependent upon impaired Hippo signaling we could not remove Sd function, as was done in the eye disc, since Sd is required for normal wing disc growth through association with the wing determination factor Vestigial [42,43]. Therefore, we utilized a *yki *null allele, *yki^B5^*, to halve the gene dosage of *yki *in a *scrib^1^*/*scrib^3 ^*mutant background. Consistent with previous reports [35], although giant larvae were still formed throughout an extended larval phase of development, wing disc overgrowth was dramatically reduced, resulting in significantly smaller wing discs with disorganized morphology (Figure 4F). In contrast, halving the dosage of *Ras85D*, a gene also essential for cell growth, proliferation and viability, did not significantly reduce wing tumor size in *scrib^1^*/*scrib^3 ^*larvae (Figure 4G), thus implicating a key role for Yki in promoting tumor overgrowth. Furthermore, halving the gene dosage of *bsk *similarly failed to rescue the overgrown phenotype of the wing discs (Figure 4H), indicating that Bsk levels, unlike Yki levels, are not rate limiting for tumor overgrowth. Thus, loss of *scrib *in the wing disc promotes tissue overgrowth through downregulation of Hippo pathway signaling, and, as in the eye disc, this is likely to be, at least in part, independent of JNK.

### Ras and Raf-driven neoplastic overgrowth of s*crib *mutants is also Sd and Yki-dependent

Loss of *scrib* also promotes tumorigenesis in cooperation with oncogenic Ras signaling [reviewed in [28]]. In a "two hit" *Drosophila *tumorigenesis model we have previously shown that although *scrib *mutant eye clones die via JNK-mediated apoptosis, if *Ras^ACT ^*or its downstream effector, *Raf^gof^*, is expressed in the mutant clones, cell death is prevented and massive and invasive tumors develop throughout an extended larval stage [39]. To determine if downregulation of Hippo pathway signaling is also an important mediator of these overgrowths we examined the expression of the Hippo pathway reporters, *ex-lacZ *and *fj-lacZ*, in *scrib^- ^*+ *Raf^gof ^*tumors.

**Figure 5:**
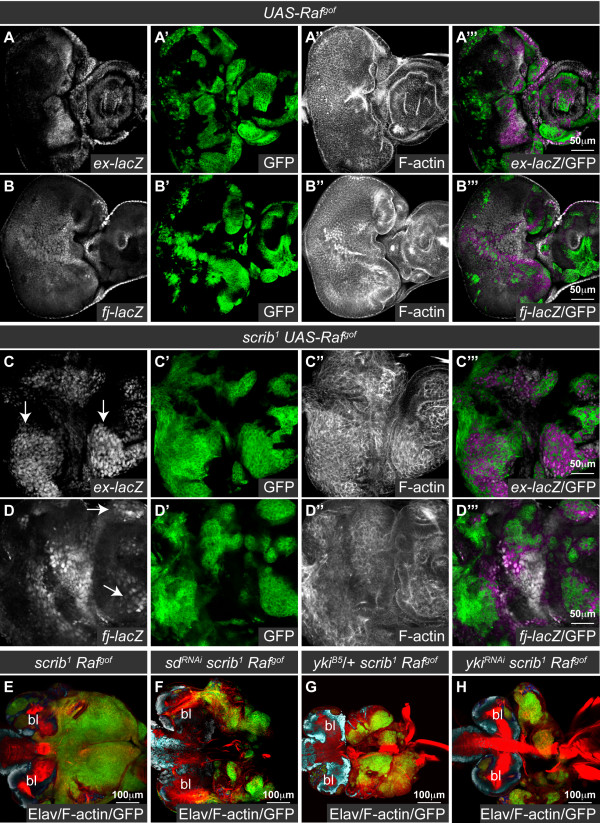
**Impaired Hippo pathway signaling promotes *scrib^1 ^*+ *Raf^gof ^*neoplastic tumor overgrowth**. Confocal sections through larval eye/antennal discs at day 5 (A-D) and eye/antennal discs still attached to the brain lobes (bl) at day 8 after egg laying (E-H). Mutant clones were generated with *ey-FLP *and are positively marked by GFP expression (green, or, in the merges, magenta in A-D and yellow in E-H). Grayscale is β-GAL (A-D), F-actin (A-D) or Elav (E-H). Red is F-actin (E-H). (A, B) Expression of *Raf^gof ^*does not alter the normal pattern of *ex-lacZ *(A) or *fj-lacZ *(B) expression in the eye disc. (C-D) *scrib^1 ^*+ *Raf^gof ^*tumors ectopically express *ex-lacZ *(C; arrows) and *fj-lacZ *(D; arrows) within the eye disc. (E-H) *scrib^1 ^*+ *Raf^gof ^*tumors overgrow and fuse with the brain lobes throughout an extended larval stage of development (E). Knockdown of *sd *(F), halving the gene dosage of *yki *(G) or expression of *yki^RNAi ^*(H) reduces *scrib^1 ^*+ *Raf^gof ^*tumor overgrowth.

The expression of *ex-lacZ *and *fj-lacZ *was predominantly unperturbed by *Raf^gof^*-expression alone (Figure 5A, B), however, as was observed by the loss of *scrib*, in *scrib^- ^*+ *Raf^gof ^*tumors both *ex-lacZ *and *fj-lacZ *expression levels were elevated (Figure 5C, D). These data thus indicated that Hippo signaling remained impaired in the tumor and could therefore be contributing to tumor growth. To determine what role the loss of Hippo signaling played in promoting tumorigenesis, we reduced Sd activity by expressing *sd^RNAi ^*in *scrib^- ^*+ *Raf^gof ^*tumors. Examination of tumors at day 5, suggested that knockdown of *sd *exerted a mild effect on reducing tumor growth (data not shown), however, pupation was not restored and the tumors continued to grow throughout an extended larval phase of development. By day 8, however, *sd^RNAi^*-expressing tumors were significantly reduced in size compared to *scrib^- ^*+ *Raf^gof ^*controls (Figure 5E, F), thus highlighting a role for reduced Hippo pathway signaling in promoting tumor overgrowth. To further confirm the importance of impaired Hippo signaling in *scrib^- ^*+ *Raf^gof ^*tumorigenesis, we also halved the gene dosage of *yki *in the tumor background, and knocked down *yki *function within the tumor using a *yki^RNAi ^*transgene [5]. Consistent with the effects of *sd^RNAi^*, both methods of limiting *yki *function were unable to restore pupation or prevent tumor overgrowth throughout an extended larval phase of development, however, they significantly reduced tumor size (Figure 5G, H). Similar reductions in tumor overgrowth were observed when *yki^RNAi ^*was expressed in *scrib^- ^*+ *Ras^ACT ^*tumors (see additional file 6). Despite the reduction in tumor overgrowth, however, knockdown of *yki *was unable to prevent tumor cells from adopting an invasive morphology and appearing to move between the brain lobes, as has been previously described [38], indicating that the tumor cells retained invasive capabilities (see additional file 6). Thus, similar to homozygous *scrib *mutants, impaired Hippo signaling promotes Ras-driven cooperative tumor overgrowth, although Yki-Sd activity is not critically rate limiting for the failure of the tumor-bearing larvae to pupate, or for the invasive nature of the tumors.

### The *scrib *mutant defects in Hippo signaling are aPKC-dependent, and aPKC signaling is sufficient to impair Hippo signaling even when JNK signaling is blocked

**Figure 6:**
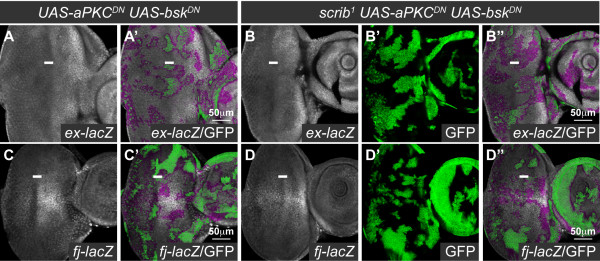
**aPKC signaling is required for the impaired Hippo pathway signaling in *scrib *mutants**. Larval eye/antennal discs with *ey-FLP *induced mutant clones marked by GFP expression (green, or magenta in the merges). Grayscale is β-GAL. A white bar indicates the location of the MF. (A-D) Coexpression of *aPKC^CAAXDN ^*and *bsk^DN ^*in clones does not alter *ex-lacZ *(A) or *fj-lacZ *(C) expression, but when *aPKC^CAAXDN ^*and *bsk^DN ^*are coexpressed in *scrib^1 ^*clones, the normal pattern of *ex-lacZ *(B) and *fj-lacZ *(D) expression is restored (compared with *scrib^1 ^*clones expressing *bsk^DN ^*alone in Figure 1).

We have shown how loss of *scrib *impairs Hippo signaling to promote tissue overgrowth in *scrib *mutant eye disc clones, in homozygous *scrib *mutant wing discs and in cooperation with oncogenic Ras-Raf signaling. As we have also demonstrated that the impaired Hippo signaling in *scrib *mutants could not be rescued by reducing JNK activity, we next sought to determine what other pathways downstream of *scrib *could be responsible for influencing Hippo signaling. Previous work in the eye disc had shown that the increased cell proliferation posterior to the MF and alterations in cell morphology in *scrib *mutant clones could both be rescued by the expression of a membrane-tethered, kinase-dead (dominant negative) form of aPKC (aPKC^CAAXDN^), although this was not sufficient to rescue the mutant cells from JNK-dependent cell death [38]. Therefore to determine if the *scrib *mutant defects in Hippo pathway activity were aPKC-dependent, we examined *ex-lacZ *and *fj-lacZ *in *scrib *mutant clones expressing *aPKC^CAAXDN^*, that were also protected from apoptosis by the coexpression of *bsk^DN^*. Strikingly, whilst the coexpression of *aPKC^CAAXDN ^*and *bsk^DN ^*in otherwise wild type clones did not alter the normal levels of *ex-lacZ *and *fj-lacZ *expression (Figure 6A, C), when both transgenes were expressed in *scrib *mutant tissue, the ectopic expression of these reporters was significantly abrogated (Figure 6B, D).

**Figure 7:**
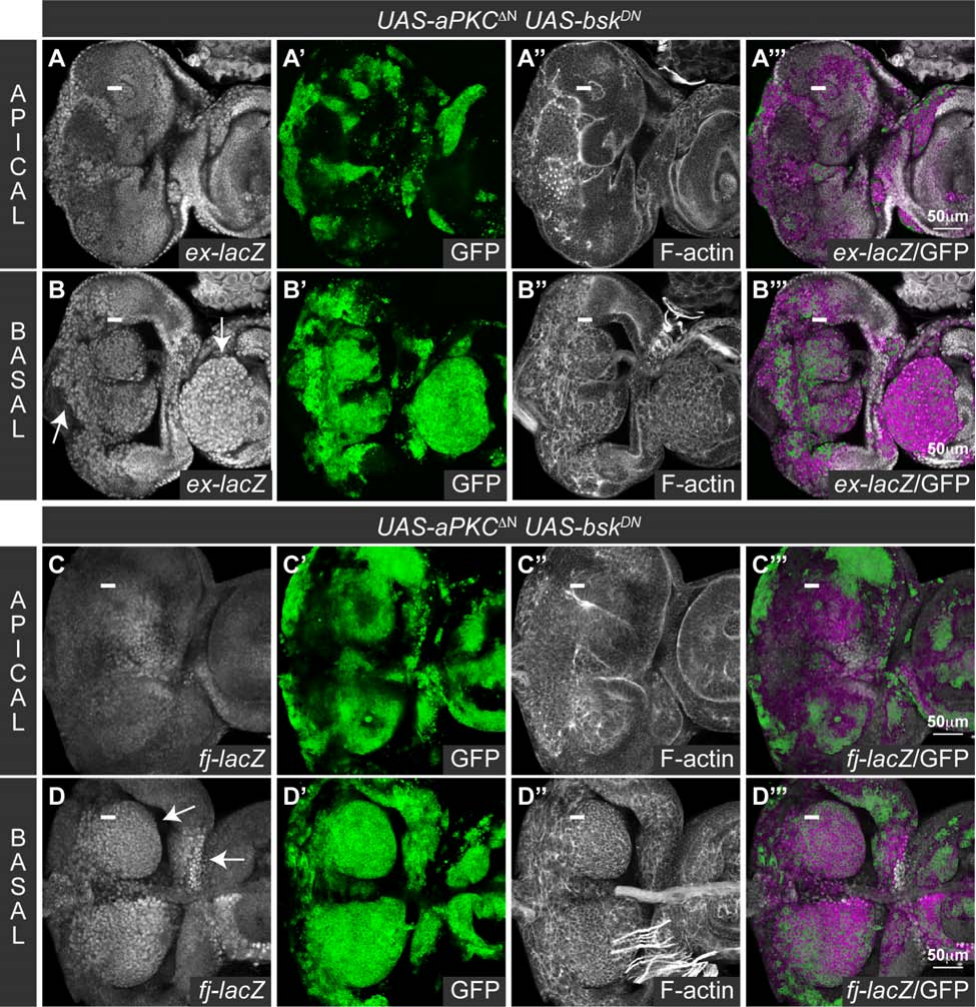
**Ectopic aPKC signaling impairs Hippo pathway signaling even when JNK signaling is blocked**. Larval eye/antennal discs with *ey-FLP *induced mutant clones marked by GFP expression (green, or magenta in the merges). Grayscale is β-GAL and F-actin. A white bar indicates the location of the MF. Apical (A, C) and basal (B, D) confocal sections are shown. (A-D) Coexpression of *aPKC^ΔN ^*with *bsk^DN ^*in clones results in large masses of tissue extruded basally from the epithelium which ectopically express *ex-lacZ *(A, B; arrows) and *fj-lacZ *(C, D; arrows).

Consistent with the *scrib *mutant defects in Hippo signaling being aPKC-dependent, ectopic activation of aPKC has been shown to be sufficient to impair Hippo signaling [35,37]. This has been linked to a capacity for aPKC to activate JNK [37], however, we have previously shown that although the expression of a truncated activated allele of *aPKC* (*aPKC^ΔN^*) in eye disc clones induces JNK-dependent cell death, if JNK signaling is blocked, ectopic cell proliferation still ensues [38]. Indeed, the Hippo pathway reporters *fj-lacZ *and *ex-lacZ* were ectopically expressed in *aPKC^ΔN ^*and *bsk^DN ^*coexpressing eye disc clones (Figure 7A-D). Thus, aPKC signaling is not only required, but is also sufficient to impair the Hippo pathway in eye disc clones in a manner that is not dependent upon JNK activity.

### The Scrib-aPKC polarity module is not a core component of the Hippo pathway

**Figure 8:**
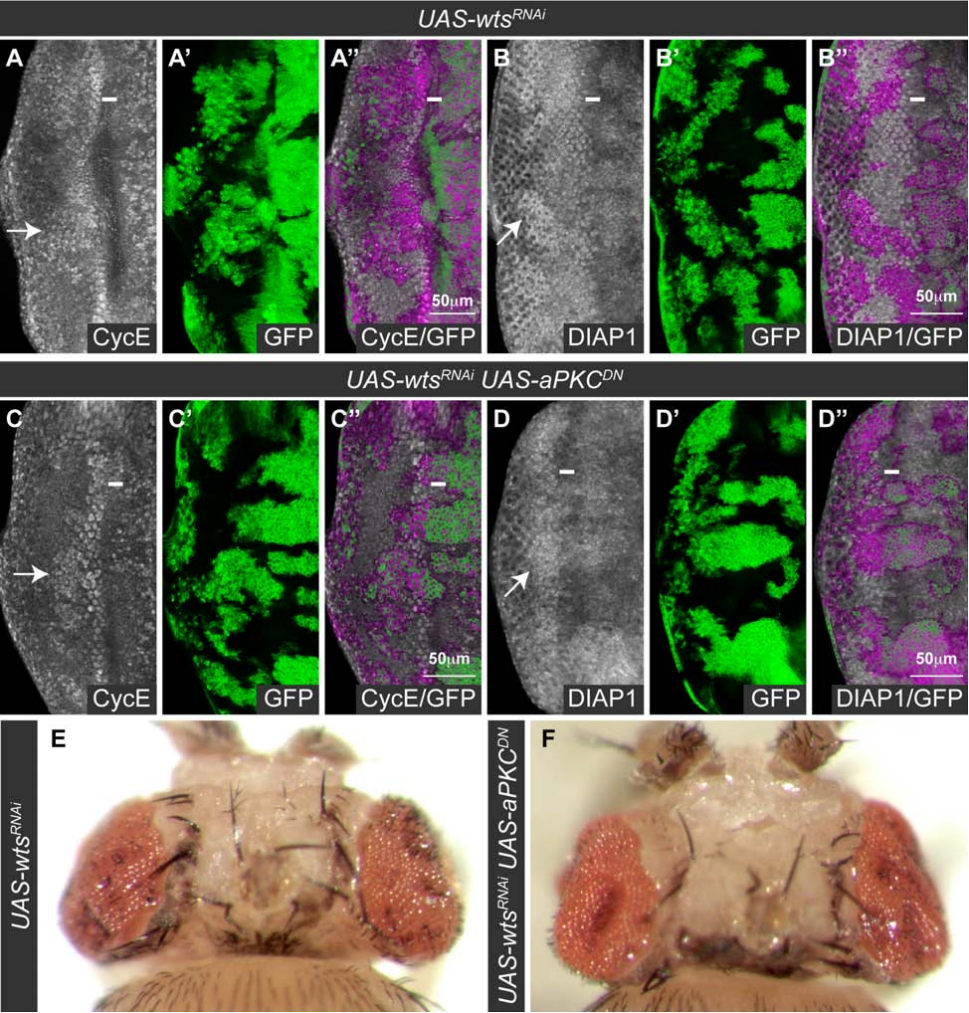
***wts *mutant overgrowth is independent of aPKC signaling**. Larval eye discs (A-D) with *ey-FLP *induced mutant clones (green, or magenta in the merges) and dorsal views of adult mosaic flies (E, F). Grayscale is CycE (A, C) and DIAP1 (B, D). A white bar indicates the location of the MF. (A-F) Expression of *wts^RNAi ^*in eye disc clones results in upregulation of CycE (A) and DIAP1 (B) posterior to the MF (example clones highlighted with arrows), resulting in adult flies eclosing with overgrown eyes (E). Coexpression of *aPKC^CAAXDN ^*with *wts^RNAi ^*does not prevent upregulation of CycE (C) and DIAP1 (D) in mutant clones (example clones highlighted with arrows), and adult flies still eclose with overgrown eyes (F).

Having demonstrated that *scrib *mutants showed an aPKC-dependent impairment to Hippo signaling, we next wanted to determine if aPKC signaling was also important for promoting tissue overgrowth in core Hippo pathway mutants such as *wts *that are known to upregulate aPKC protein levels [30,31]. However, although *wts^RNAi^*-expressing clones ectopically express CycE and DIAP1 and overgrow (Figure 8A, B), similar to *scrib *mutant clones, coexpression of *aPKC^CAAXDN ^*in *wts^RNAi ^*clones neither prevented the ectopic expression of CycE or DIAP1 (Figure 8C, D), nor suppressed the resulting overgrown adult eye phenotype (Figure 8E, F). Consistent with this, knockdown of *aPKC* by RNAi (to levels at which the protein was significantly reduced; see additional file 7), or overexpression of *scrib *or *lgl* was insufficient to restrain *wts^RNAi^*-mediated overgrowth (see additional file 8). Similar conclusions were obtained with analyzing the more upstream Hippo pathway component, *ft*. Neither *aPKC^CAAXDN ^*overexpression (see additional file 9), nor the overexpression of a constitutively active allele of *lgl*, *lgl^3A ^*(data not shown), could prevent the ectopic expression of CycE in *ft^RNAi^*-expressing clones. Thus, aPKC signaling is not required to promote either *wts *or *ft *mutant tissue overgrowth.

Although these data indicated that aPKC signaling was not important for *wts *or *ft *mutant overgrowth, it was still possible that under normal conditions, when Hippo pathway activity functions to inhibit tissue overgrowth, endogenous levels of Hippo pathway activity could be susceptible to an aPKC-mediated restraint. However, knockdown of *aPKC* by RNAi in both the eye disc and the wing disc did not decrease DIAP1 levels (data not shown) or reduce *fj-lacZ *or *ex-lacZ *expression, as would be expected upon hyper-activation of the Hippo pathway (see additional file 10). Furthermore, overexpression of *scrib* or *lgl* in the wing disc was also insufficient to increase Hippo pathway activation and reduce *fj-lacZ *or *ex-lacZ *expression (see additional file 10). We thus conclude that during normal tissue growth, endogenous levels of aPKC activity are not required to critically restrain Hippo pathway activity, and nor are the overexpression of *scrib* or *lgl *sufficient to ectopically activate the pathway. Thus the aPKC-Scrib polarity module does not function as a core component of the Hippo pathway, and is only likely to impinge upon Hippo signaling during specific developmental or pathological contexts.

### Loss of *scrib *impairs Hippo pathway signaling downstream, or in parallel to, *expanded *and *fat*

**Figure 9:**
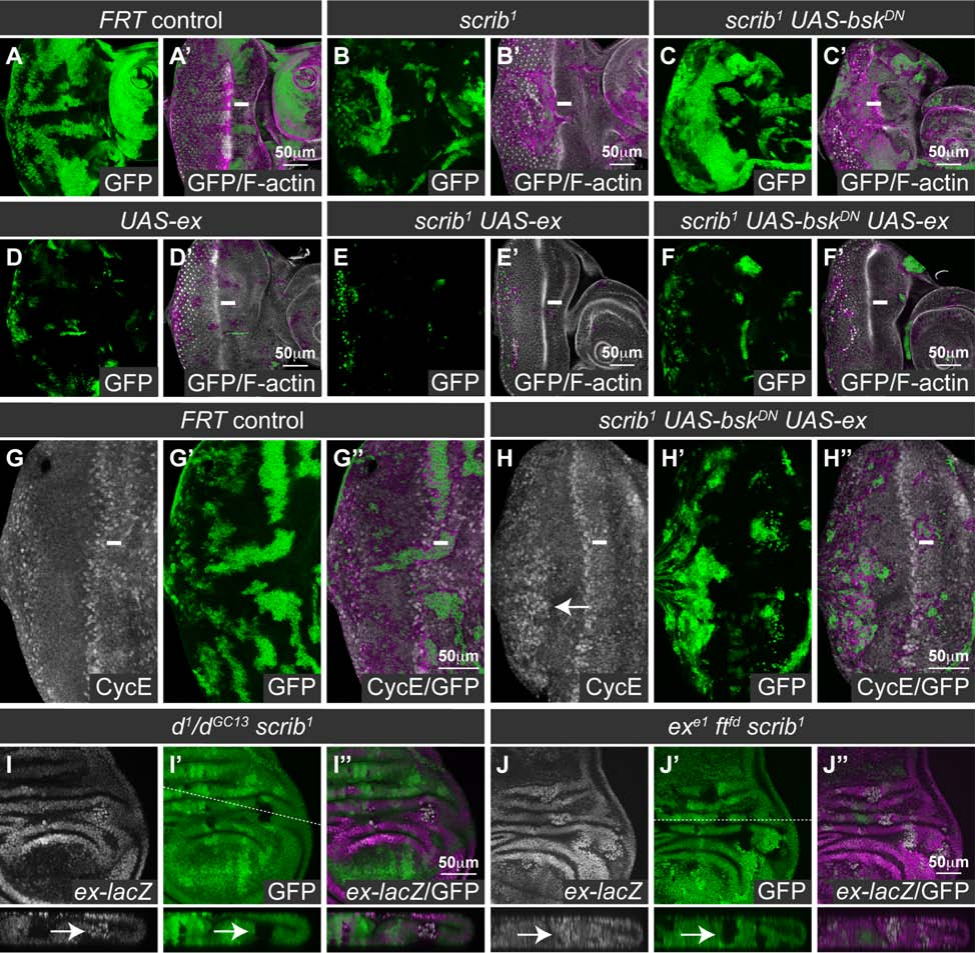
**Loss of *scrib *impairs Hippo pathway signaling in parallel to *expanded *and *fat***. Larval eye/antennal disc clones generated with *ey-FLP *and marked by GFP expression (A-H), and wing disc clones generated by *hs-FLP *and marked by the absence of GFP (I, J). Green is GFP (or magenta in the merges). Grayscale is F-actin (A-F), CycE (G, H) and β-GAL (I, J). A white bar indicates the location of the MF in the eye disc (A-H), and a dashed white line marks the location of the Z-stacks shown below the wing discs (I, J). (A-C) *scrib^1 ^*clones (B) are reduced in size compared to control clones (A), but expressing *bsk^DN ^*in the *scrib^1 ^*clones dramatically increases clone size (C). (D-F) Overexpression of *ex *in otherwise wild type clones reduces clonal tissue size (D), and when *ex *is expressed in *scrib^1 ^*(E), or *scrib^1 ^*clones expressing *bsk^DN ^*(F), clonal tissue size is also greatly reduced. (G, H) Overexpression of *ex *in *scrib^1 ^*clones expressing *bsk^DN ^*does not prevent ectopic expression of CycE in basally located mutant clones posterior to the MF (H, highlighted with arrow), as compared to control clones (G). (I, J) *scrib^1 ^*clones generated in the wing disc (arrows) in either a transheterozygous *d*^*1*^/*d*^*GC13*^mutant background (I) or homozygous *ft^fd ^ex^e1 ^*(J) mutant background show increased *ex-lacZ *compared to the surrounding tissue.

To gain insight into how aPKC-dependent deregulation of the Hippo pathway occurs in *scrib *mutants we carried out overexpression and epistasis experiments with different Hippo pathway components. Upstream regulation of Hippo signaling occurs through at least two transmembrane proteins, Ft and Crb [reviewed in [44]]. Crb acts through Ex [32-34], and Ft acts through both Ex [14,15,19] and the unconventional myosin Dachs (D) [12]. The overexpression of *ex *can promote ectopic Hippo signaling, and when expressed in otherwise wild type clones can restrain clonal growth in a Hpo and Wts-dependent manner [13,17,41]. Significantly, the overexpression of *ex *in *scrib *mutant eye disc clones also restrained clonal growth, and this was the case even if JNK-mediated apoptosis of the clonal tissue was prevented (Figure 9A-F). Indeed, *scrib *clones expressing *bsk^DN ^*result in pupal lethality, but the coexpression of *ex *was sufficient to rescue some flies to adult viability (data not shown). This indicated that in the absence of *scrib*, Ex was still capable of inducing Hippo pathway activation, and suggested that the Hippo pathway was affected in *scrib *mutants upstream or in parallel to Ex. However, despite the reduced clonal growth of *scrib *mutant cells expressing *ex *and *bsk^DN^*, the mutant clones were still larger than *ex*-expressing clones alone, and the mutant tissue could still clearly be seen to ectopically express CycE posterior to the MF (Figure 9G, H). This suggested that the overexpression of *ex *was not able to fully inhibit Yki activation in *scrib *mutants and that the loss of *scrib *can, at least in part, impair Hippo pathway signaling downstream of, or in parallel to, *ex*.

**Figure 10:**
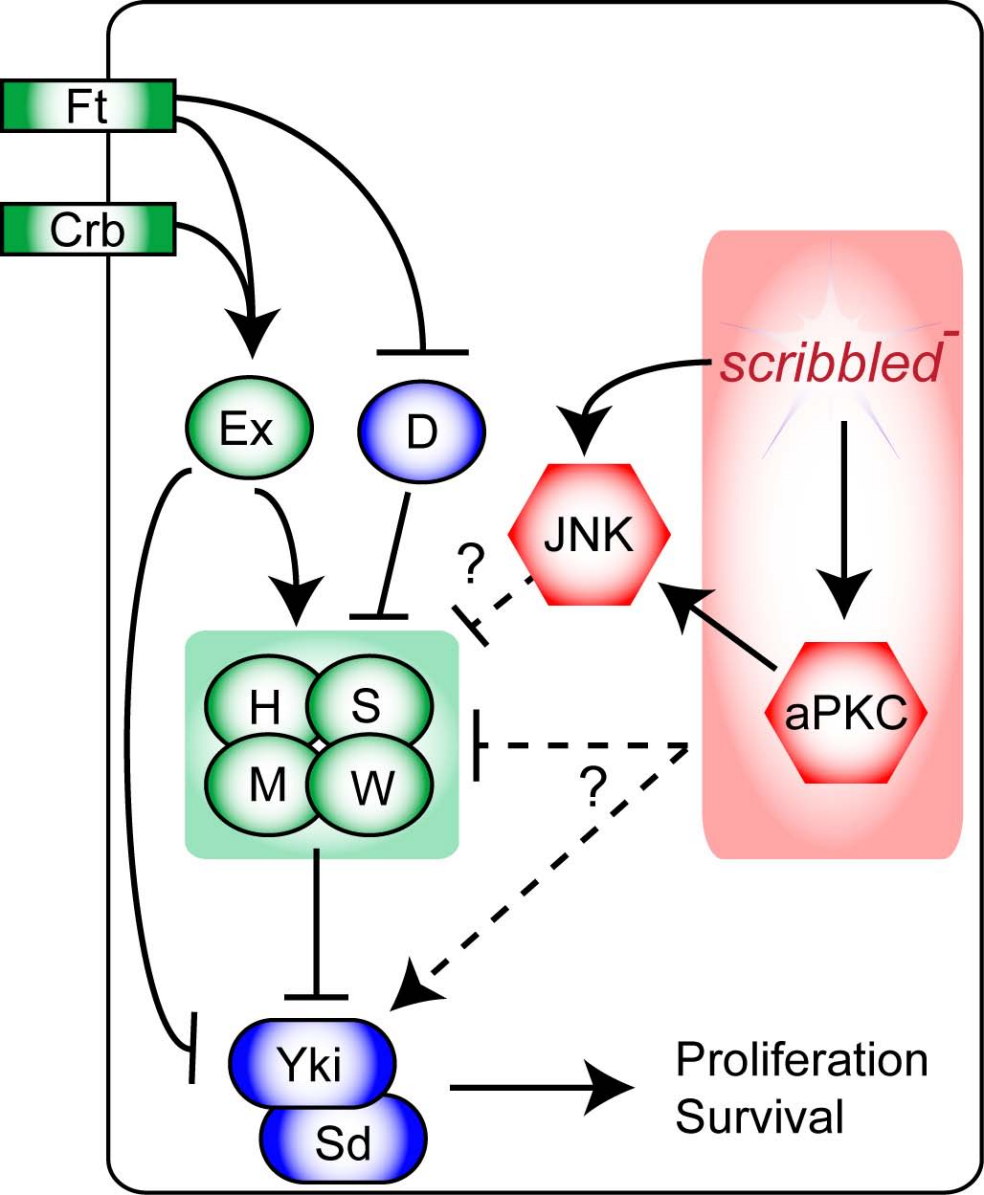
**Model for how the loss of *scrib *affects Hippo pathway signaling**. In an epithelial cell, the core Hippo pathway components Hpo (H), Wts (W), Sav (S) and Mats (M) inhibit the activity of Yki, which acts to regulate transcription through its DNA binding partner, Sd. Extracellular regulation of the pathway occurs through the two transmembrane proteins, Crb acting through Ex, and Ft, acting through both Ex and D. Ex can also directly bind to and inhibit Yki. Loss of *scrib *in the eye disc is associated with aPKC-dependent inhibition of the Hippo pathway. Although JNK signaling is activated by either the loss of *scrib*, or ectopic aPKC activity, JNK is not required for Hippo pathway inhibition. In the wing disc, loss of *scrib *is also associated with JNK activation, and although JNK signaling has been shown to be sufficient to downregulate the Hippo pathway in the wing disc [37], Hippo pathway signaling remains impaired in *scrib *mutants even when JNK is blocked. Epistasis experiments suggest that pathway inhibition in *scrib *mutants occurs, at least in part, downstream or in parallel to Ex and Ft, and could impact upon either the core components (H/S/M/W) or upon Yki activity itself.

Ft can act in parallel to Ex, and indeed the overexpression of *ex *in *ft *mutant clones is unable to restrain *ft *mutant tissue overgrowth [16]. We therefore next determined if impaired Ft signaling could be responsible for downregulating the Hippo pathway in *scrib *mutants. To do this we generated *scrib *clones in a *d *mutant animal background, since Ft acts through inhibiting D and loss of *d *fully abrogates *ft *mutant overgrowth [12] (see Figure 10). Interestingly, however, even in the absence of *d *we could still detect upregulation of the Hippo pathway reporter *ex-lacZ *in *scrib *mutant clones (Figure 9I), suggesting that loss of *scrib *also impaired Hippo signaling downstream of, or in parallel to, Ft-D. As it was possible that in fact both the Ft and Ex arms of Hippo pathway regulation were impaired in *scrib *mutants we next generated *scrib *clones in a *ft ex *double mutant animal background. Significantly, *ex-lacZ *expression was once again hyper-activated in *scrib *clones when compared to the basal level of pathway deregulation observed in the tissue surrounding the mutant clones that was mutant for *ft *and *ex *alone (Figure 9J). Thus, together with the *ex *overexpression analysis showing a degree of Hippo pathway impairment that could not be blocked by increasing Ex levels, the data is consistent with the loss of *scrib *impairing Hippo pathway signaling downstream, or in parallel to, both Ft and Ex (Figure 10).

## Discussion

Tumorigenesis may be considered as an abnormality in organogenesis and tissue regeneration, during which tissue growth is not restrained by signals that would normally function to restrict organ size. The Hippo pathway is a potent force in restraining organ overgrowth, and it is therefore logical that such a control mechanism would be perturbed during the unrestrained overgrowth of neoplasias. Indeed, in this study we show that loss of the neoplastic tumor suppressor *scrib *promotes tissue overgrowth through downregulation of the Hippo pathway. Furthermore, loss of *scrib *sensitizes cells to neoplastic transformation by *Ras^ACT^*/*Raf^gof^*, and reduced Hippo signaling cooperates with Ras-Raf to promote tumor overgrowth. Thus, impaired Hippo signaling is clearly a key force in driving *scrib *mutant tissue overgrowth. It is pertinent to note, however, that whilst knockdown of *yki *was able to completely abrogate the overgrowth of *wts *mutant clones (see additional file 3), it was not able to fully rescue tumor overgrowth of *scrib*^- ^+ *Ras^ACT ^*tumors throughout an extended larval stage of development. Thus, other deregulated pathways in *scrib *mutants are likely to also be important for promoting tumor overgrowth. Indeed, knockdown of *yki *also failed to rescue the loss of apico-basal cell polarity in *scrib *mutants, the capacity of *scrib*^- ^+ *Ras^ACT ^*tumor cells to invade, and the failure of the *scrib*^- ^+ *Ras^ACT ^*tumor-bearing larvae to pupate. Although we cannot exclude the possibility that further reducing Yki activity would more effectively rescue these tumor phenotypes, the data indicate that Yki levels are not critically rate limiting for the polarity and invasive properties of *scrib *mutant cells.

### How does *scrib *regulate the Hippo pathway?

The impairment to Hippo signaling in *scrib *mutants was significantly rescued by reducing aPKC activity. As loss of *scrib *is also associated with aPKC-dependent apico-basal cell polarity defects, it initially seemed probable that the Hippo pathway might be affected in *scrib *mutants through the deregulation of apically localized Hippo pathway receptors. Indeed, in zebrafish Scrib binds to, and functionally cooperates with, Ft [45]; and as this interaction could be conserved in *Drosophila *[45] very direct points of intersection between Scrib and the upstream regulators of Hippo signaling can be envisaged. Our work does not rule out this possibility, and in fact, some of our data suggest that Hippo signaling is perturbed upstream of *ex *since *ex *overexpression significantly restrained *scrib *mutant tissue overgrowth. However, this interpretation is complicated by Ex's capacity to directly bind to, and sequester Yki activity [46]. In fact, even *ex *overexpression was not sufficient to block ectopic CycE expression in *scrib *mutant clones, and epistasis experiments confirm that the deregulated Hippo signaling in *scrib *mutants is at least partially epistatic to both *ex *and *ft*, and thus downstream of two known transmembrane proteins that regulate the pathway, Crb and Ft (Figure 10). Nevertheless, despite the evidence placing *scrib *downstream of these proteins, there is likely to be a close relationship between Scrib's role as a cell polarity regulator and impaired Hippo signaling. Indeed, a number of recent reports indicating that increased levels of F-actin are sufficient to inhibit Hippo pathway activity [47,48] are also suggestive, since the aPKC-dependent loss of apico-basal cell polarity in *scrib *mutants is often associated with F-actin accumulations (data not shown). Thus, it is possible that a number of mechanisms, including receptor mislocalization and F-actin accumulation, might be operative in driving Hippo pathway impairment when apico-basal cell polarity is disrupted in *scrib *mutants. Interestingly, however, *lgl *mutant eye disc clones are not associated with the severe alterations in cell morphology characteristic of *scrib *mutant cells, yet the impairment to Hippo signaling in *lgl *mutant clones is also dependent upon aPKC activity [35]. Furthermore, Ex and Ft localization were unaffected in the absence of *lgl*, whilst both Hpo and Ras-associated domain family protein (RASSF) were co-mislocalized, consistent with the Hippo pathway being impaired downstream of Ft and Ex [35]. Whether a common aPKC-dependent mechanism of Hippo pathway inhibition is operative in both *lgl *and *scrib *mutant eye discs will require further investigation. We note, however, that this regulation is not likely to reflect a core, rate limiting role for Scrib-Lgl in promoting, or aPKC in repressing, Hippo pathway activity since neither overexpressing Scrib or Lgl, nor knockdown of aPKC, led to significant Hippo pathway activation. Furthermore, even though the expression of an activated form of aPKC could induce the expression of Hippo pathway reporters, and thus downregulate the pathway, neither the overexpression of a wild type, membrane-tethered aPKC in eye disc clones (data not shown), nor clones of hypomorphic *scrib *alleles [35, and data not shown], consistently increased DIAP1 or CycE levels. The ability of cells to accommodate such wide fluctuations in Scrib, Lgl and aPKC activity suggests that aPKC-dependent Hippo pathway inhibition may only occur during specific developmental or pathological contexts during which, for instance, a threshold level is reached whereby either compromised Scrib-Lgl activity can no longer correctly localize or restrain aPKC-mediated inhibition of the pathway, or increased aPKC levels can no longer be restrained by normal levels of Scrib-Lgl. Alternative models placing Scrib-Lgl downstream of aPKC, and thus involving an aPKC-mediated impairment to Hippo pathway signaling through inhibition of Scrib-Lgl function, also remain possible and need to be considered.

### The role of JNK in Hippo pathway regulation, and differences between loss of *scrib *and *lgl *in the wing disc

A recent report indicates that the impaired Hippo pathway signaling in *lgl *mutant wing discs is dependent upon JNK signaling, and that ectopic aPKC signaling in the wing disc also acts through JNK to promote Yki activity [37]. In the eye disc, however, we show that Hippo pathway impairment in *scrib *mutants, as well as upon ectopic activation of aPKC signaling, cannot be rescued by blocking JNK, and this is also likely to be the case for *lgl *mutants [49]. Furthermore, in *scrib *mutant wing discs, although we demonstrate that JNK signaling is ectopically activated upon *scrib *knockdown, we also show that Hippo pathway impairment in the wing occurs, at least in part, even when JNK signaling is blocked. Why loss of *scrib*, unlike loss of *lgl *in the wing disc, does not require JNK activation to impair Hippo signaling is not yet clear. In *lgl *mutants, the cell polarity defects in the wing disc are also JNK-dependent [37], and while we did not analyze cell polarity markers upon *scrib *knockdown in the wing, one possibility is that loss of *scrib *is associated with JNK-independent cell polarity defects that impact upon the Hippo pathway in a similar manner to the eye disc. Certainly loss of *scrib *is notable for eliciting much stronger cell morphology defects in the eye disc than loss of *lgl *[39,50]. As the cell morphology defects in *scrib *mutant eye disc clones are aPKC-dependent [38], it will be important to determine what role aPKC signaling plays in the *scrib *mutant wing disc phenotypes.

Although our data indicates that JNK is not required for Hippo pathway impairment in *scrib *mutants, it does not rule out the possibility that JNK signaling still contributes to enhance Hippo pathway deregulation. Furthermore, clearly JNK plays many other important roles in neoplastic progression, including roles in promoting invasion and a failure to pupate. Indeed, the role of JNK in neoplasia is proving to be complex, since in some contexts it functions as an oncogene to promote neoplasia, whilst in different contexts it acts as a tumor suppressor through the induction of apoptosis [38,39,51-53]. Its effects upon Hippo pathway regulation may therefore also be context dependent. In fact, the activation of Hippo pathway reporters in *scrib *mutant clones in the eye disc (in which JNK is known to be activated) were often variable, some cells clearly showing ectopic expression of DIAP1, *ex-lacZ *and *fj-lacZ*, whilst other mutant cells did not upregulate these reporters. Non-cell autonomous effects, with increased expression in wild type cells surrounding mutant clones, were also sometimes observed (data not shown), consistent with the involvement of the Hippo pathway in regenerative proliferation around dying tissue [54]. Much of this variability appeared to be reduced when JNK signaling was blocked in the mutant clones, and whilst the rescue of some non-cell autonomous effects would be consistent with the role that JNK can play in promoting non-cell autonomous compensatory proliferation through Yki activity [37], it also remains possible that JNK may act in opposite ways to downregulate DIAP1 or other reporters in mutant cells destined to die. Further analysis will be required to decipher how cellular context defines the dual functions of JNK as tumor suppressor or oncogene, and how this is related to Hippo pathway regulation.

### Emerging cross talk between the Hippo pathway and regulators of tissue architecture

Extensive cross talk is beginning to emerge between the Hippo pathway and regulators of cell morphology. Genes involved in controlling epithelial apico-basal cell polarity and levels of F-actin can impact upon Hippo pathway signaling, but reciprocally impaired Hippo signaling can affect cell morphology pathways. Loss of *wts *is capable of eliciting neoplastic overgrowth [55], and Hippo pathway mutants induce apical hypertrophy with increased levels of apical cell determinants such as Crb, aPKC [30,31], and F-actin [48], although, interestingly, despite the potential for increased Crb, aPKC and F-actin to promote tissue hyperplasia, the overgrowth in Hippo pathway mutants is independent of the apical hypertrophy [30,31]. This is consistent with our own work demonstrating that *wts *and *ft *mutant tissue overgrowth was independent of aPKC signaling. Furthermore, the interrelationship between Hippo and apico-basal cell polarity pathways is not confined to traditional apico-basal epithelial polarity regulators such as Scrib and aPKC, since the Hippo pathway components *ft, fj *and *ds *also participate in planar cell polarity pathways [reviewed in [56]], as do *scrib *[57], *lgl *[58] and *aPKC *[59,60]. The emerging picture is therefore one of a complex network of interactions whereby multiple components regulating cellular architecture are employed by cells to read their position within a morphogenetic field and respond with appropriate Hippo-pathway regulated tissue growth.

## Conclusions

In summary, this work demonstrates that loss of *scrib *results in epithelial tissue overgrowth in both the eye and wing discs through downregulation of the Hippo pathway. Thus Scrib joins an increasing number of epithelial apico-basal cell polarity regulators that have links with Hippo pathway control. Whether these connections are operative in mammals is not yet clear, however, both the Hippo organ size control and Scribble cell polarity function are highly conserved. Loss of Hippo pathway signaling or ectopic activation of the Yki or Sd homologues, (YAP and TEAD family proteins respectively), is emerging as a powerful oncogenic force [reviewed in [21]]. Similarly, the mammalian Scrib module is increasingly implicated in tumorigenesis [reviewed in [28]], and mammalian Scrib can restrain tissue transformation by oncogenic Ras [61], and Myc [62]. Mammalian Scrib also functions within planar cell polarity pathways [63], and although links with the Hippo pathway have not yet been described, the connection is likely to be conserved since studies in the zebrafish indicate that zScrib binds to zFat1 and also promotes Hippo pathway activation [44]. The uniting of these two powerful tumor suppressor pathways clearly has important implications for human carcinogenesis. Loss of apico-basal cell polarity is considered to be a critical hallmark of neoplastic transformation, and it will be important to determine if in mammalian cancer this is also associated with defective Hippo signaling and subsequent tumor overgrowth.

## Methods

### *Drosophila *stocks

The following *Drosophila *stocks were used: *ey-FLP1, UAS-mCD8-GFP;;Tub-GAL4, FRT82B, Tub-GAL80 *[64]; *y, w, hs-FLP*; *FRT82B, Ubi-GFP*; *en-GAL4*; *UAS-DaPKC^ΔN ^*[26]; *UAS-DaPKC^CAAXDN ^*[65]; *UAS-aPKC^RNAi ^*(VDRC #2907); *bsk^2 ^*[66]; *UAS-bsk^DN ^*[67]; *UAS-bsk^RNAi ^*(NIG #5680R-1); *d^1 ^*[68]; *d^GC13 ^*[12]; *ex^e1 ^*[69]; *ex^697 ^*(*ex-lacZ *in all figures except Figure 9J which is *ex^e1^*) [69]; *UAS-ex *[70]; *ft^fd ^*[71]; *UAS-ft^RNAi ^*(VDRC #9396); *fj-lacZ *[72]; *UAS-lgl^5.1 ^*[26]; *UAS-lgl^3A ^*[26]; *UAS-phl^gof ^*(*UAS-Raf^gof^*) [73]; *Ras85D^e1b ^*[74]; *UAS-dRas1^V12 ^*[75]*; UAS-scrib^19.2 ^*[38]; *FRT82B, scrib^1 ^*[76]; *scrib^3 ^*[77]; *UAS-scrib^RNAi ^*(VDRC #27424); *UAS-sd^RNAi ^*(NIG #8544R-2); *wts^X1 ^*[78]; *UAS-wts^RNAi ^*(NIG #12072R-1); *yki^B5 ^*[7]; *UAS-yki^RNAi ^*[5].

### Mosaic analysis

Clonal analysis utilized either MARCM (mosaic analysis with repressible cell marker) [79] with *FRT82B *and *ey*-*FLP1 *to induce clones and *mCD8-GFP *expression to mark mutant tissue, or for negatively marked *scrib^1 ^*clones, *hs-FLP *with *FRT82B, Ubi-GFP*. All fly crosses were carried out at 25°C and grown on standard fly media. Heat shock clones were induced by a temperature shift to 37°C for 15 minutes, and discs were harvested at 64 hours after clone induction. For the examination of *scrib^1 ^*clones in a *d *mutant background, *hs-FLP *induced *FRT82B, scrib^1 ^*clones were generated in a *d^1^/d^GC13^, ex^697^, FRT40A *mutant background. For the examination of *scrib^1 ^*clones in a *ft ex *double mutant background, *hs-FLP *induced *FRT82B, scrib^1 ^*clones were generated in a *ft^fd^, ex^e1^, FRT40A *homozygous mutant background.

### Immunohistochemistry

Imaginal discs were dissected in phosphate-buffered saline (PBS) from either wandering 3^rd ^instar larvae or from staged lays of larvae for genotypes which failed to pupate and entered an extended larval stage of development. Tissues were fixed in 4% formaldehyde in PBS, and blocked in 2% goat serum in PBT (PBS 0.1% Triton X-100). For the detection of S phase cells, a 1 h BrdU pulse at 25°C was followed by fixation, immuno-detection of GFP, further fixation, acid treatment and immuno-detection of the BrdU epitope. Primary antibodies were incubated with the samples in block overnight at 4°C, and were used at the following concentrations; mouse anti-β-galactosidase (Rockland) at 1 in 400, mouse anti-Elav (Developmental Studies Hybridoma Bank) at 1 in 20, rat anti-Cyclin E (Helen McNeill) at 1 in 400, mouse anti-DIAP1 (Bruce Hay) at 1 in 100, rabbit anti-GFP (Invitrogen) at 1 in 1000, mouse anti-BrdU (Becton-Dickinson) at 1 in 50. Secondary antibodies used were; anti-mouse/rat Alexa647 (Invitrogen) and anti-rabbit Alexa488 (Invitrogen) at 1 in 400. F-actin was detected with phalloidin-tetramethylrhodamine isothiocyanate (TRITC; Sigma, 0.3 μM) at 1 in 1000. Samples were mounted in 80% glycerol.

### Microscopy and image processing

All samples were analyzed by confocal microscopy on an Olympus FV1000 microscope. Single optical sections were selected in Flouroview^® ^software before being processed in Adobe Photoshop^®^CS2 and assembled into figures in Adobe Illustrator^®^CS2.

## List of Abbreviations

aPKC: atypical protein kinase C; BrdU: bromodeoxyuridine; BSA: bovine serum albumin; bsk: basket; crb: crumbs; cycE: cyclin E; d: dachs; DIAP1: *Drosophila *inhibitor of apoptosis 1; dlg: discs large; DN: dominant negative; ds: dachsous; ey: eyeless; ex: expanded; fj: four-jointed; FRT: flippase recognition target; hpo: hippo; JNK: Jun N-terminal kinase; lgl: lethal giant larvae; MARCM: mosaic analysis with repressible marker; MF: morphogenetic furrow; PBS: phosphate-buffered saline; sav: salvador; scrib: scribbled; sd: scalloped; wts: warts; yki: yorkie.

## Authors' contributions

KD carried out experiments for Figure 5 and Additional file 2 and 7, interpreted data and contributed editorial guidance. FAG carried out epistasis experiments for Figure 9, interpreted data and contributed editorial guidance. HER interpreted data and contributed editorial guidance. AMB conceived of the study, designed and carried out all other experiments, interpreted data, and wrote the paper. All authors read and approved of the final manuscript.

## Supplementary Material

Additional file 1 ***scrib *mutant cells in the eye disc exhibit impaired Hippo pathway signaling**. Confocal sections through 3^rd ^instar larval eye/antennal discs (A-F), posterior to the left. Control clones generated using a wild type chromosome with a *Flippase recognition target *(*FRT*) (A, C, E) and *scrib^1 ^*clones (B, D, F) were generated with *ey-FLP *and are positively marked by GFP expression (green, or magenta in the merges). Grayscale is DIAP1 (A-B), β-GAL (C-F), and F-actin (A-F). A white bar indicates the location of the MF. (A-B) Compared to control clones (A), DIAP1 levels in *scrib^1 ^*clones (B) are variable, low in some cells (arrowhead) and higher in others (arrow). (C-F) In *scrib^1 ^*clones, *ex-lacZ *is ectopically expressed in some clones (D; arrow), but not in all mutant cells (D; arrowhead), and *fj-lacZ *expression is similarly upregulated in some (F; arrow), but not all, mutant clones. Click here for file

Additional file 2 ***scrib *mutant cells expressing *bsk^RNAi ^*have impaired Hippo pathway signaling**. Larval eye/antennal discs with clones marked by GFP expression (green, or magenta in the merges). Grayscale is BrdU (A), β-Gal (B) and F-actin (B). A white bar indicates the location of the MF. (A, B) *scrib^1 ^*clones expressing *bsk^RNAi ^*exhibit ectopic BrdU incorporation posterior to the MF (A), similar to *scrib^1 ^*cells expressing *bsk^DN ^*(see Figure 2B), and ectopic expression of *fj-lacZ *within the mutant tissue (B; arrow). Click here for file

Additional file 3 **Knockdown of *sd *and *yki *rescues *wts *mutant overgrowth**. Larval eye/antennal discs (A-D) with clones marked by GFP expression (green, or magenta in the merges), and dorsal views of adult mosaic flies (E, F). Grayscale is BrdU (A, B) and β-Gal (C, D). A white bar indicates the location of the MF. (A, B) *wts^X1 ^*mutant clones ectopically proliferate posterior to the MF (A), however, expression of *sd^RNAi ^*in *wts^X1 ^*mutant clones restores the normal pattern of cell proliferation (B). (C, D) The ectopic expression of *fj-lacZ *in *wts^X1 ^*mutant clones (C) is reduced, although not completely normalized, by *sd^RNAi ^*expression in the mutant clones (D; compare to C in which the expression extends to the edges of the disc). (E, F) Expressing *sd^RNAi ^*in *wts^X1 ^*mutant clones rescues *wts^X1^*-mediated pupal lethality, and adult flies eclose with normal sized, although slightly roughened, adult eyes (E). The expression of *yki^RNAi ^*in *wts^X1 ^*mutant eye disc clones (F) produces a similar rescue of eye disc overgrowth and adult fly viability as *sd^RNAi^*. Click here for file

Additional file 4 **Knockdown of *sd *normalizes *fj-lacZ *expression in *scrib *mutant clones**. Larval eye/antennal disc clones marked by GFP (green, or magenta in the merges). The location of the MF is indicated by a white bar. Grayscale is β-Gal. (A-C) Expression of *sd^RNAi ^*in clones does not alter the normal pattern of *fj-lacZ *expression (A), but when *sd^RNAi ^*is expressed in *scrib^1 ^*clones (B), or *scrib^1 ^*clones expressing *bsk^DN ^*(C), it prevents ectopic *fj-lacZ *expression in the mutant tissue (compare to Figure 1F). Click here for file

Additional file 5 **Expression of *scrib^RNAi ^*reduces Scrib protein levels**. Confocal section through a 3^rd ^instar larval wing disc. *en-GAL4 *driven expression of *scrib^RNAi ^*in the posterior half of the disc is marked by GFP expression (green, or magenta in the merges). Grayscale is Scrib and F-actin. (A) *en-GAL4 *driven expression of *scrib^RNAi ^*greatly reduces Scrib protein levels and results in abnormalities in tissue morphology, as observed by F-actin. Click here for file

Additional file 6 **Knockdown of *yki *reduces Ras-driven tumor overgrowth, although tumor cells retain invasive capabilities**. Pairs of larval eye/antennal discs attached to the brain lobes at day 10, with mutant tissue generated by *ey-FLP *and marked by GFP expression (green, or yellow in the merges). Red is F-actin. (A-C) *scrib^1 ^*+ *Ras^ACT ^*tumors are massively overgrown and fused together (A), but knockdown of *yki *within the tumors significantly restrains tumor overgrowth (B), although tumor cells are still observed moving between the brain lobes (arrows) indicating that they retain invasive capabilities (C). Click here for file

Additional file 7 **Expression of *aPKC^RNAi ^*reduces aPKC protein levels**. Larval eye/antennal disc with mutant tissue generated by *ey-FLP *and marked by GFP expression (green, or magenta in the merges). Grayscale is aPKC and F-actin. (A) Expression of *aPKC^RNAi ^*in clones greatly reduces aPKC protein levels, although tissue morphology, as observed by F-actin, remains relatively unperturbed. Click here for file

Additional file 8 **Ectopic cell proliferation in *wts^RNAi ^*clones is rescued by *sd *knockdown, but not by decreasing aPKC or increasing Lgl-Scrib levels**. Dorsal views of adult mosaic flies (A, B, D-F), and larvae eye disc clones (C) generated by *ey-FLP *and marked by GFP (green, or magenta in the merges) and CycE (grayscale). A white bar indicates the location of the MF. (A-B) Expression of *sd^RNAi ^*with *wts^RNAi ^*rescues the *wts^RNAi^*-dependent overgrown adult eye phenotype (A), but knockdown of aPKC does not rescue *wts *mutant overgrowth (B; see Figure 8E for a comparison of *wts^RNAi^*-expressing adult flies). (C, D) Expression of an allele of *lgl *that can't be inactivated by aPKC phosphorylation *(lgl^3A^)* neither prevents ectopic CycE expression posterior to the MF (C; arrow), nor rescues the overgrown adult eye phenotype (D) of *wts^RNAi^*-expressing clones. (E-F) Overexpression of wild type versions of *lgl* (E) or *scrib* (F) do not rescue the *wts^RNAi ^*overgrown adult eye phenotype. Click here for file

Additional file 9 **Ectopic cell proliferation in *ft^RNAi^*-expressing clones cannot be rescued by *aPKC^DN ^*expression.** Larval eye discs with GFP-expressing mutant clones (green, or magenta in the merges) induced by *ey-FLP*. Grayscale is CycE, and the MF is indicated by a white bar. (A, B) *ft^RNAi^*-expressing clones ectopically express CycE posterior to the MF (A; arrow), and this is not blocked by the coexpression of *aPKC^CAAXDN ^*(B; arrow). Click here for file

Additional file 10 **Endogenous aPKC signaling does not limit Hippo pathway activity.** Larval eye discs (A, B) with *ey-FLP *induced clones, and wing discs (C-F) with *en-GAL4 *driven expression of transgenes (green, or magenta in the merges). Grayscale is β-GAL (A-F), and F-actin (A, B). A white bar indicates the location of the MF in the eye discs. (A-D) Expression of *aPKC^RNAi ^*in eye disc clones or in the wing disc does not reduce *ex-lacZ *(A, C) or *fj-lacZ *(B, D) expression, in fact *ex-lacZ *expression in the wing disc is slightly elevated. (E, F) The normal pattern of *fj-lacZ *expression in the wing disc is not altered by overexpression of wild type versions of *scrib* (E) or *lgl *(F). Click here for file
