# Supplementary figures and images for: Loss of the Drosophila cell polarity regulator Scribbled promotes epithelial tissue overgrowth and cooperation with oncogenic Ras-Raf through impaired Hippo pathway signaling
Source: BMC Dev Biol. 2011 Sep 29;11:57. doi: 10.1186/1471-213X-11-57 (PMC3206446; doi:10.1186/1471-213X-11-57)

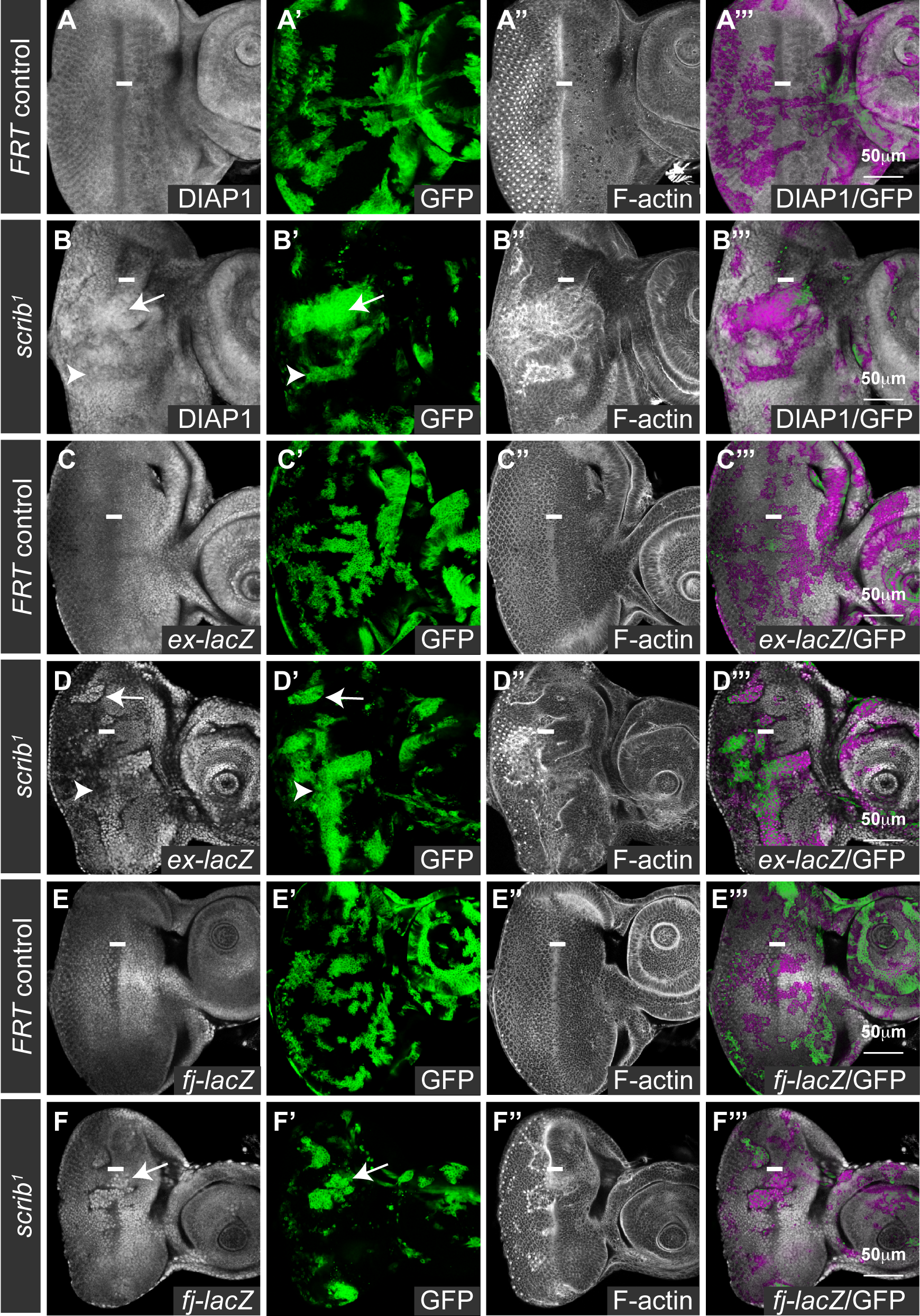

Supplement: Additional file 1 — scrib mutant cells in the eye disc exhibit impaired Hippo pathway signaling. Confocal sections through 3rd instar larval eye/antennal discs (A-F), posterior to the left. Control clones generated using a wild type chromosome with a Flippase recognition target (FRT) (A, C, E) and scrib1 clones (B, D, F) were generated with ey-FLP and are positively marked by GFP expression (green, or magenta in the merges). Grayscale is DIAP1 (A-B), β-GAL (C-F), and F-actin (A-F). A white bar indicates the location of the MF. (A-B) Compared to control clones (A), DIAP1 levels in scrib1 clones (B) are variable, low in some cells (arrowhead) and higher in others (arrow). (C-F) In scrib1 clones, ex-lacZ is ectopically expressed in some clones (D; arrow), but not in all mutant cells (D; arrowhead), and fj-lacZ expression is similarly upregulated in some (F; arrow), but not all, mutant clones. [file 1471-213X-11-57-S1.TIFF]

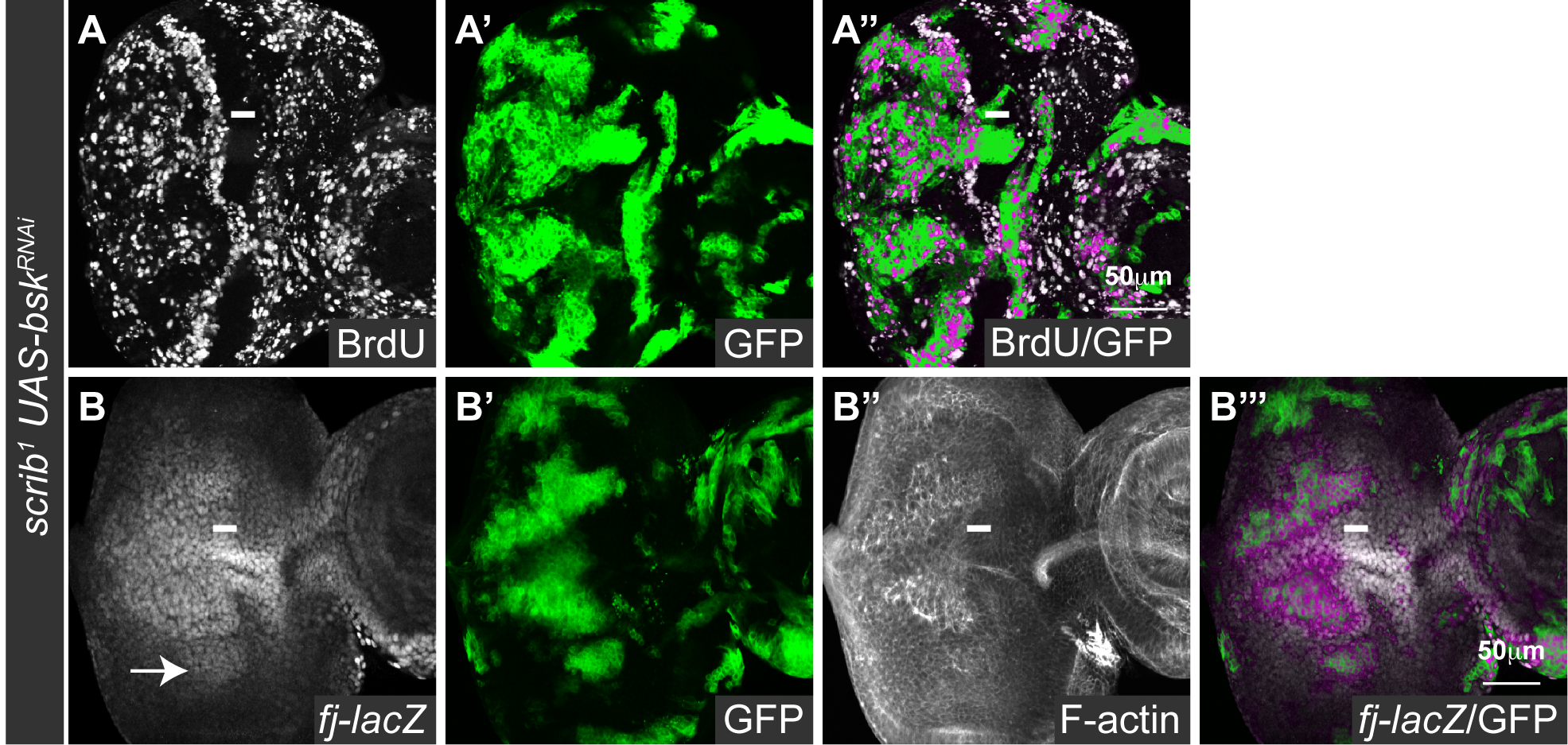

Supplement: Additional file 2 — scrib mutant cells expressing bskRNAi have impaired Hippo pathway signaling. Larval eye/antennal discs with clones marked by GFP expression (green, or magenta in the merges). Grayscale is BrdU (A), β-Gal (B) and F-actin (B). A white bar indicates the location of the MF. (A, B) scrib1 clones expressing bskRNAi exhibit ectopic BrdU incorporation posterior to the MF (A), similar to scrib1 cells expressing bskDN (see Figure 2B), and ectopic expression of fj-lacZ within the mutant tissue (B; arrow). [file 1471-213X-11-57-S2.TIFF]

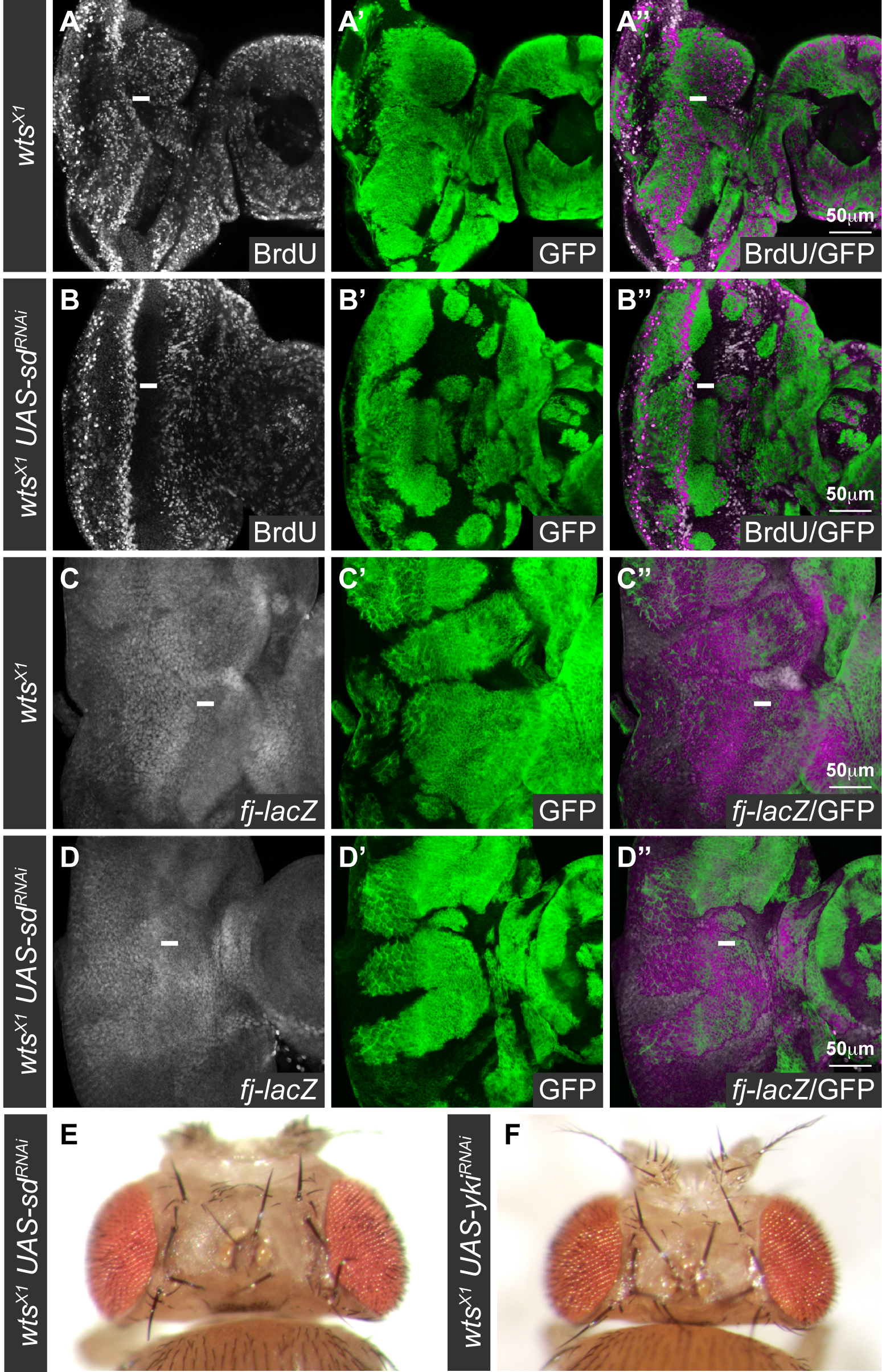

Supplement: Additional file 3 — Knockdown of sd and yki rescues wts mutant overgrowth. Larval eye/antennal discs (A-D) with clones marked by GFP expression (green, or magenta in the merges), and dorsal views of adult mosaic flies (E, F). Grayscale is BrdU (A, B) and β-Gal (C, D). A white bar indicates the location of the MF. (A, B) wtsX1 mutant clones ectopically proliferate posterior to the MF (A), however, expression of sdRNAi in wtsX1 mutant clones restores the normal pattern of cell proliferation (B). (C, D) The ectopic expression of fj-lacZ in wtsX1 mutant clones (C) is reduced, although not completely normalized, by sdRNAi expression in the mutant clones (D; compare to C in which the expression extends to the edges of the disc). (E, F) Expressing sdRNAi in wtsX1 mutant clones rescues wtsX1-mediated pupal lethality, and adult flies eclose with normal sized, although slightly roughened, adult eyes (E). The expression of ykiRNAi in wtsX1 mutant eye disc clones (F) produces a similar rescue of eye disc overgrowth and adult fly viability as sdRNAi. [file 1471-213X-11-57-S3.TIFF]

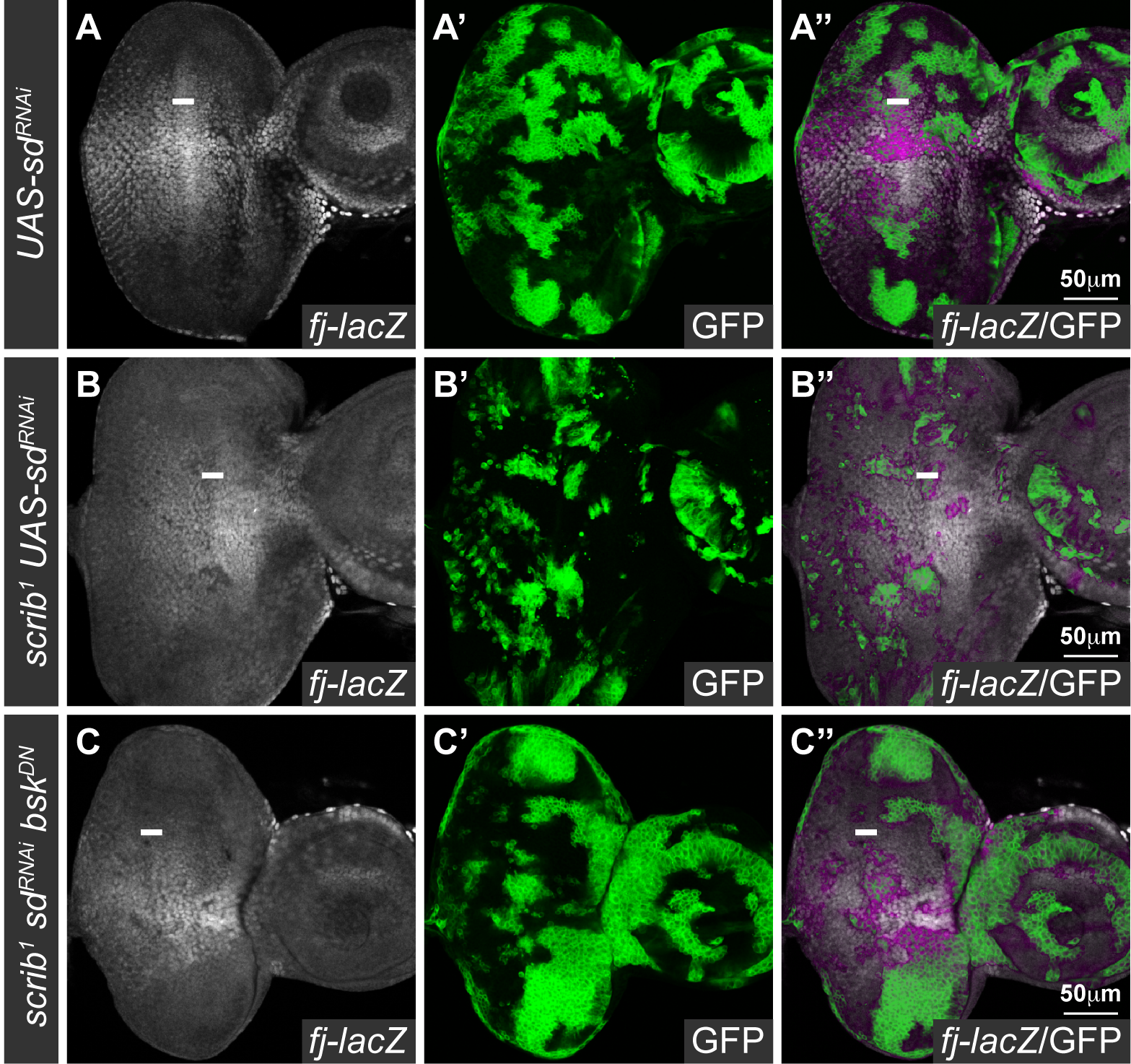

Supplement: Additional file 4 — Knockdown of sd normalizes fj-lacZ expression in scrib mutant clones. Larval eye/antennal disc clones marked by GFP (green, or magenta in the merges). The location of the MF is indicated by a white bar. Grayscale is β-Gal. (A-C) Expression of sdRNAi in clones does not alter the normal pattern of fj-lacZ expression (A), but when sdRNAi is expressed in scrib1 clones (B), or scrib1 clones expressing bskDN (C), it prevents ectopic fj-lacZ expression in the mutant tissue (compare to Figure 1F). [file 1471-213X-11-57-S4.TIFF]

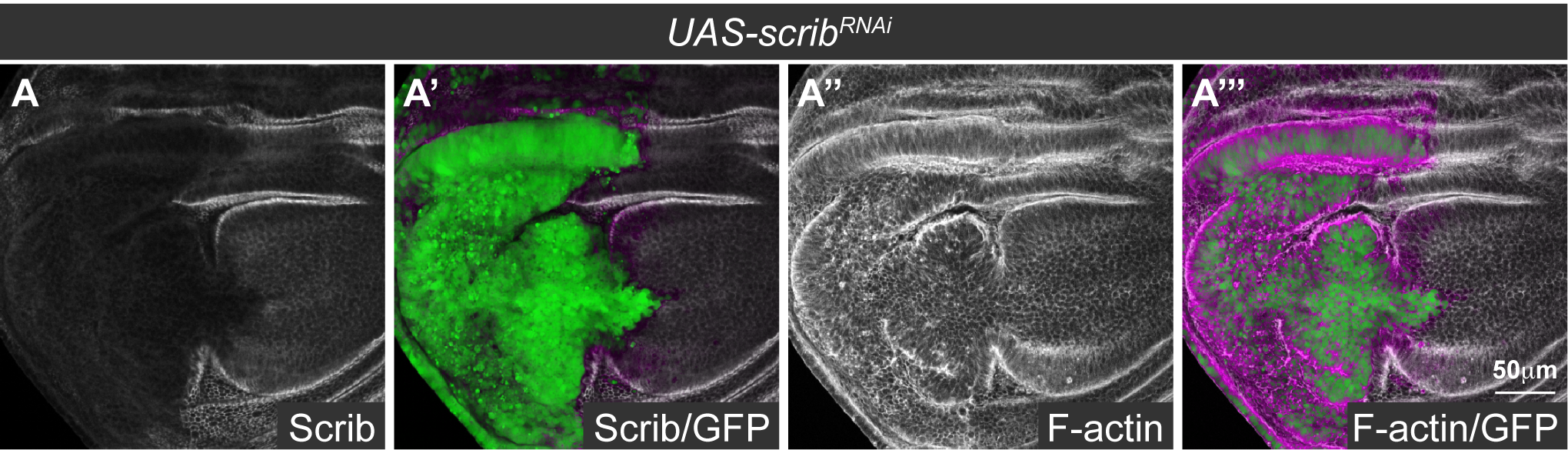

Supplement: Additional file 5 — Expression of scribRNAi reduces Scrib protein levels. Confocal section through a 3rd instar larval wing disc. en-GAL4 driven expression of scribRNAi in the posterior half of the disc is marked by GFP expression (green, or magenta in the merges). Grayscale is Scrib and F-actin. (A) en-GAL4 driven expression of scribRNAi greatly reduces Scrib protein levels and results in abnormalities in tissue morphology, as observed by F-actin. [file 1471-213X-11-57-S5.TIFF]

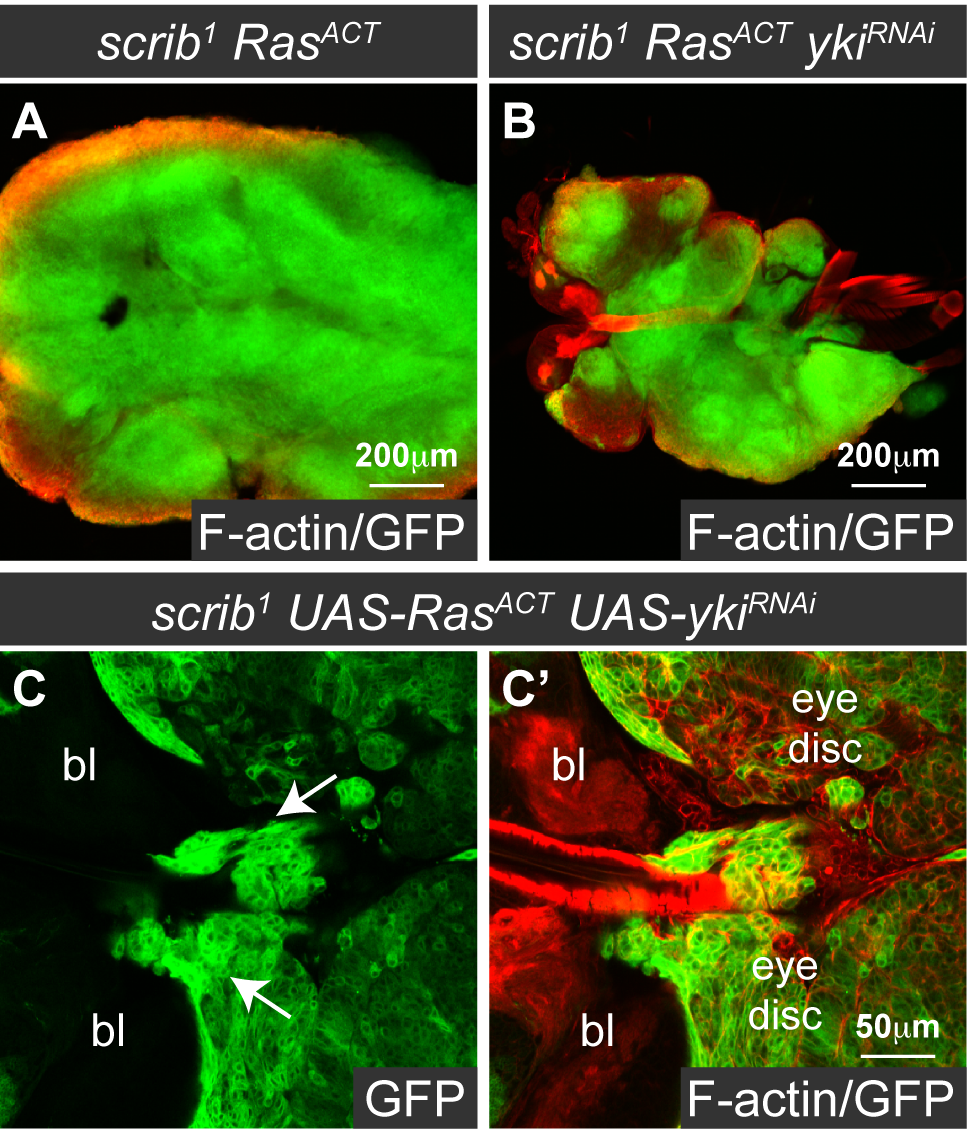

Supplement: Additional file 6 — Knockdown of yki reduces Ras-driven tumor overgrowth, although tumor cells retain invasive capabilities. Pairs of larval eye/antennal discs attached to the brain lobes at day 10, with mutant tissue generated by ey-FLP and marked by GFP expression (green, or yellow in the merges). Red is F-actin. (A-C) scrib1 + RasACT tumors are massively overgrown and fused together (A), but knockdown of yki within the tumors significantly restrains tumor overgrowth (B), although tumor cells are still observed moving between the brain lobes (arrows) indicating that they retain invasive capabilities (C). [file 1471-213X-11-57-S6.TIFF]

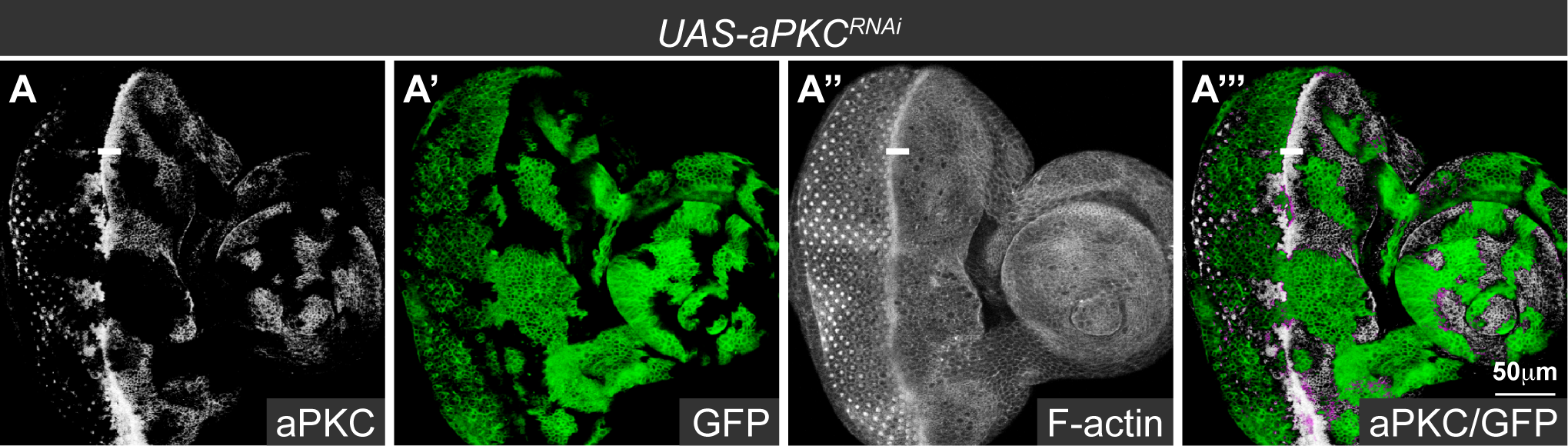

Supplement: Additional file 7 — Expression of aPKCRNAi reduces aPKC protein levels. Larval eye/antennal disc with mutant tissue generated by ey-FLP and marked by GFP expression (green, or magenta in the merges). Grayscale is aPKC and F-actin. (A) Expression of aPKCRNAi in clones greatly reduces aPKC protein levels, although tissue morphology, as observed by F-actin, remains relatively unperturbed. [file 1471-213X-11-57-S7.TIFF]

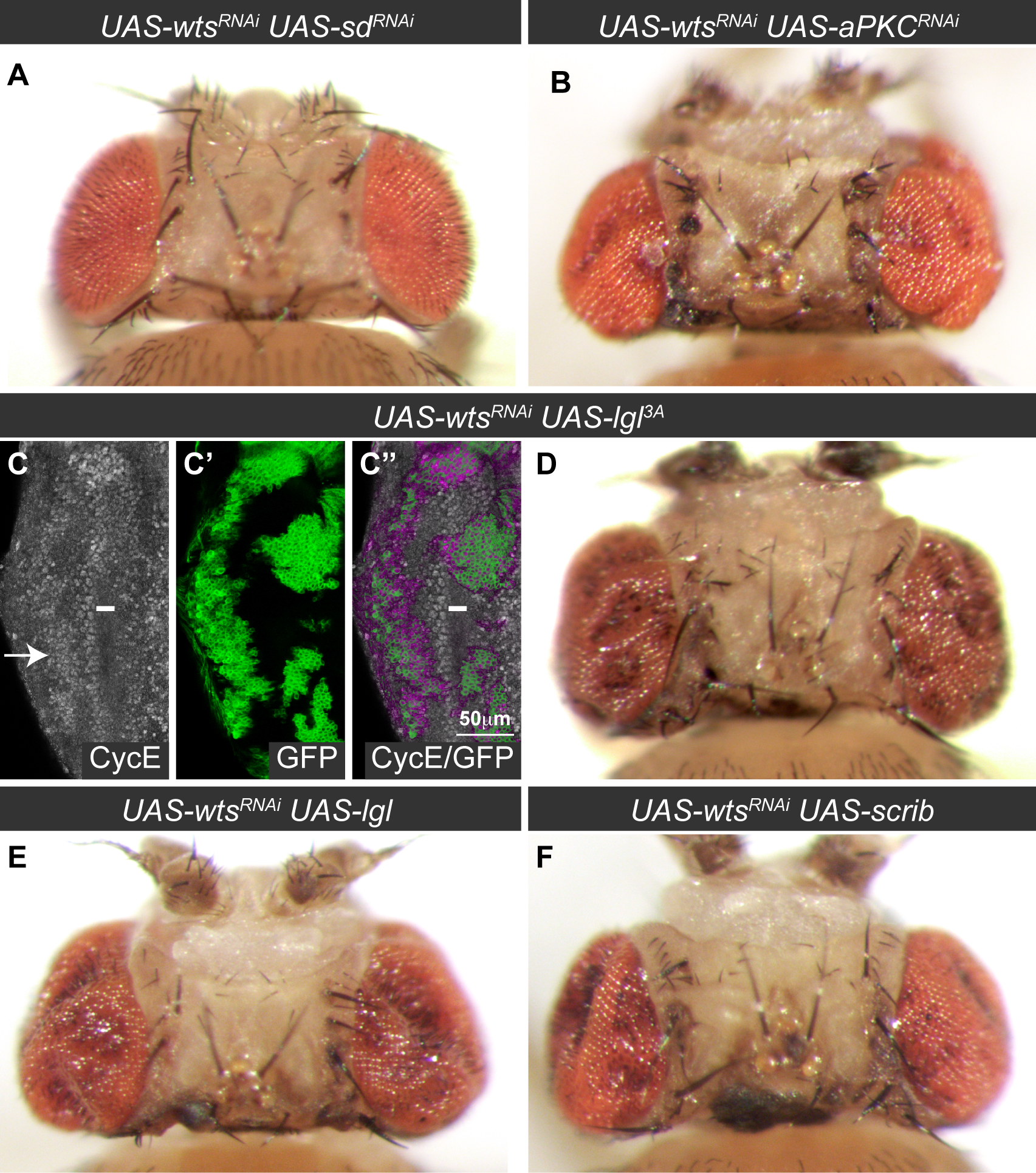

Supplement: Additional file 8 — Ectopic cell proliferation in wtsRNAi clones is rescued by sd knockdown, but not by decreasing aPKC or increasing Lgl-Scrib levels. Dorsal views of adult mosaic flies (A, B, D-F), and larvae eye disc clones (C) generated by ey-FLP and marked by GFP (green, or magenta in the merges) and CycE (grayscale). A white bar indicates the location of the MF. (A-B) Expression of sdRNAi with wtsRNAi rescues the wtsRNAi-dependent overgrown adult eye phenotype (A), but knockdown of aPKC does not rescue wts mutant overgrowth (B; see Figure 8E for a comparison of wtsRNAi-expressing adult flies). (C, D) Expression of an allele of lgl that can't be inactivated by aPKC phosphorylation (lgl3A) neither prevents ectopic CycE expression posterior to the MF (C; arrow), nor rescues the overgrown adult eye phenotype (D) of wtsRNAi-expressing clones. (E-F) Overexpression of wild type versions of lgl (E) or scrib (F) do not rescue the wtsRNAi overgrown adult eye phenotype. [file 1471-213X-11-57-S8.TIFF]

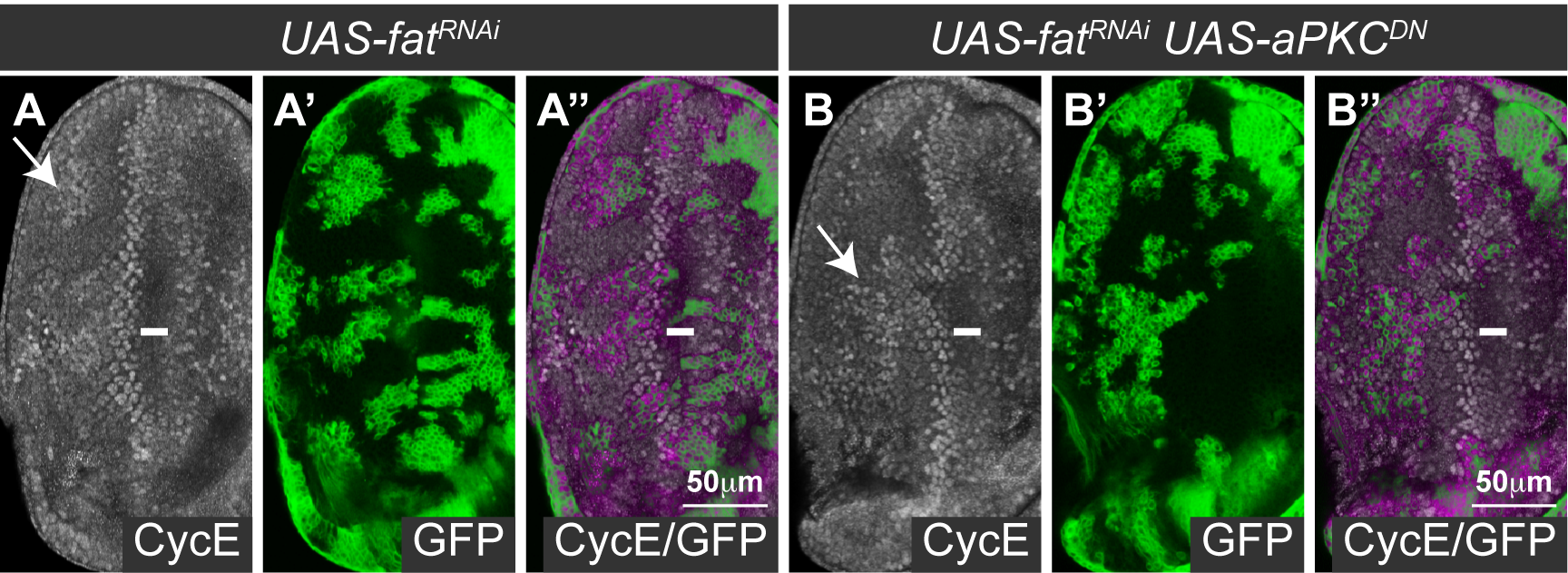

Supplement: Additional file 9 — Ectopic cell proliferation in ftRNAi-expressing clones cannot be rescued by aPKCDN expression. Larval eye discs with GFP-expressing mutant clones (green, or magenta in the merges) induced by ey-FLP. Grayscale is CycE, and the MF is indicated by a white bar. (A, B) ftRNAi-expressing clones ectopically express CycE posterior to the MF (A; arrow), and this is not blocked by the coexpression of aPKCCAAXDN (B; arrow). [file 1471-213X-11-57-S9.TIFF]

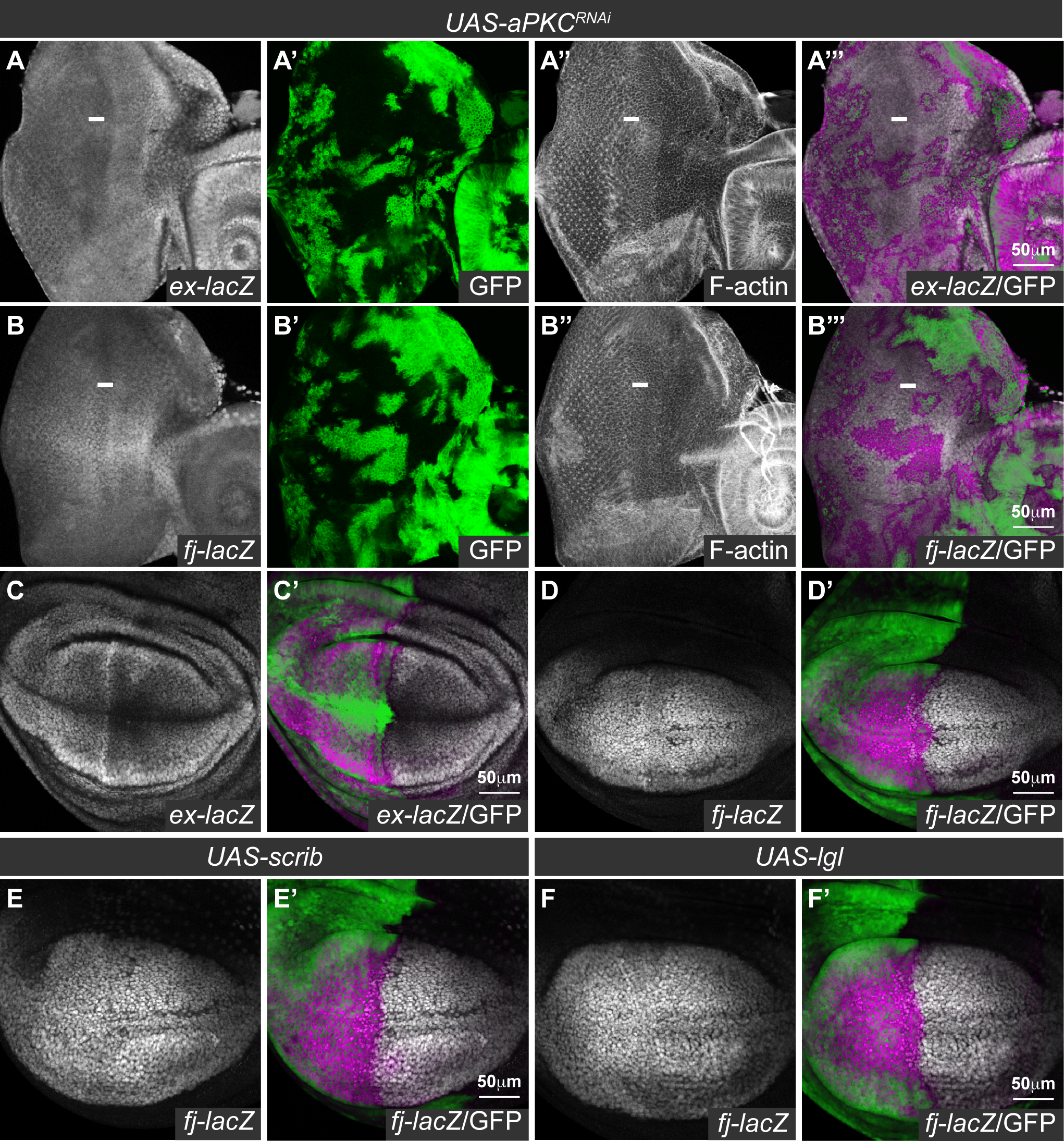

Supplement: Additional file 10 — Endogenous aPKC signaling does not limit Hippo pathway activity. Larval eye discs (A, B) with ey-FLP induced clones, and wing discs (C-F) with en-GAL4 driven expression of transgenes (green, or magenta in the merges). Grayscale is β-GAL (A-F), and F-actin (A, B). A white bar indicates the location of the MF in the eye discs. (A-D) Expression of aPKCRNAi in eye disc clones or in the wing disc does not reduce ex-lacZ (A, C) or fj-lacZ (B, D) expression, in fact ex-lacZ expression in the wing disc is slightly elevated. (E, F) The normal pattern of fj-lacZ expression in the wing disc is not altered by overexpression of wild type versions of scrib (E) or lgl (F). [file 1471-213X-11-57-S10.TIFF]
